# The progress of autoimmune hepatitis research and future challenges

**DOI:** 10.1515/med-2023-0823

**Published:** 2023-10-30

**Authors:** Yang Zhang, Dehe Zhang, Ling Chen, Jing Zhou, Binbin Ren, Haijun Chen

**Affiliations:** Graduate Department of Zhejiang Chinese Medicine University, Hangzhou, Zhejiang, China; Department of Infectious Diseases, Affiliated Jinhua Hospital, Zhejiang University School of Medicine, Jinhua, China

**Keywords:** autoimmune hepatitis, pathogenic mechanism, diagnosis, treatment, animal models

## Abstract

Autoimmune hepatitis (AIH) is a chronic liver inflammatory disease with various immune system manifestations, showing a global trend of increased prevalence. AIH is diagnosed through histological abnormalities, clinical manifestations, and biochemical indicators. The biochemical markers involve interfacial hepatitis, transaminase abnormalities, positive autoantibodies, etc. Although AIH pathogenesis is unclear, gene mutations and immunological factors could be the leading factors. AIH usually presents as a chronic liver disease and sometimes as acute hepatitis, making it challenging to distinguish it from drug-related hepatitis due to similar clinical symptoms. Normalizing transaminases and serum IgG levels is essential in assessing the remission status of AIH treatment. Glucocorticoids and azathioprine are the first-line AIH treatment, with lifelong maintenance therapy in some patients. The quality of life and survival can be improved after appropriate treatment. However, certain limitations jeopardize the quality of treatment, including long treatment cycles, side effects, poor patient compliance, and inability to inhibit liver fibrosis and cirrhosis. Accurate AIH animal models will help us understand the pathophysiology of the disease while providing fresh perspectives for avoiding and treating AIH. This review will help us understand AIH better, from the cellular and molecular causes to the clinical features, and will provide insight into new therapy techniques with fewer side effects.

## 1 Introduction

The diagnosis and treatment of non-viral hepatitis pose a significant concern in the context of chronic liver disease [1,2]. This review aims to provide a comprehensive overview of the fundamental and clinical aspects of autoimmune hepatitis (AIH), considering its insidious start, various clinical presentations, and intricate diagnostic process. AIH is a chronic inflammatory liver disease caused by hepatocytes being attacked by immune cells [3]. Circulating autoantibodies, increased hyperimmunoglobulin G and transaminases, and histological interface hepatitis characterize AIH. AIH can progress to hepatic sclerosis, liver failure, or even death without proper treatment [4]. Without efficient diagnostic biomarkers, AIH is categorized as type 1 (positive for anti-nuclear ANA, anti-smooth muscle ASM, anti-soluble liver antigen SLA, antibodies, etc.) and type 2 (anti-liver-kidney microsome antibody type 1, anti-liver cytosol type 1, anti-liver kidney microsomal type 3, etc.), based on autoantibody expression and other characteristics [5]. AIH patients are globally distributed and are predominantly female. AIH can manifest at any age or ethnicity, with most patients generally older than 40 years [6]. The prevalence of AIH is approximately 24.5 per 100,000 in the Asia-Pacific region and is significantly higher in stable populations [7,8]. AIH prevalence and clinical presentation could be associated with genetic predisposition, environmental factors, medications, and ethnicity, leading to hepatitis, decompensated cirrhosis, and liver cancer [9]. AIH severely threatens public health, seriously impacting economic and societal development.

## 2 Pathogenic mechanism

AIH causes autoimmune intolerance, resulting in autoimmune cells attacking hepatocytes [1]. However, AIH pathogenesis, which could involve genetic predisposition, environmental factors, and immune system dysregulation, remains incompletely elucidated [10,11].

### 2.1 Genetic factors

AIH is a polygenic disease of unknown etiology, and genetic factors can influence the disease progression and clinical phenotype of AIH [12]. AIH patients are associated with extrahepatic autoimmune disease, and about 40% have an autoimmune disease history in the family [13]. Genome-wide association studies have revealed that the human leukocyte antigen (HLA) locus and some non-HLA loci are primarily associated with the AIH region [12]. AIH genetic susceptibility is related to HLA region genes on the short arm of chromosome 6, particularly with DRB1 allelic variants [14]. The diagnostic effectiveness of HLA susceptibility alleles in identifying individuals with AIH is widely recognized, leading to their inclusion in the AIH diagnostic criteria [15]. MHC class II HLA DRB1 alleles DRB1*0301 and DRB1*0401 encode amino acid sequences that render humans in Europe and North America more susceptible to AIH-1 [16,17]. DRB1*15:01 can reduce the risk of AIH1 in the population [18]. Besides, DRB1*03, DRB1*07, and DQB1*02:01 will make the population more susceptible to AIH-2 [19,20]. Other genes also affect AIH development since some patients with the disease do not carry HLA alleles. In addition to non-HLA genes having immunological significance, the cytotoxic T lymphocyte-associated antigen 4 (CTLA-4) gene + 49A/G genotype was significantly correlated with AIH susceptibility [21]. The CTLA-4 gene, located on chromosome 2q33, includes a single base exchange polymorphism in exon 1, in which there is a guanine replaced by adenine at position 49, resulting in the expressed protein There is a threonine instead of alanine. A recent investigation obtained the genotypes of 155 persons diagnosed with AIH who belong to the Nordic Caucasian population and 102 healthy individuals from the same racial background. The expression of each genotype was AA = 50/155 patients vs 51/102 controls; AG = 84/155 patients vs 38/102 controls; GG = 21/155 patients vs 13/102 controls, *x*2 = 8.94, *P* = 0.011). In addition, there is a synergistic effect between CTLA-4 + 49A/G and DRB1*03:01 on AIH-1 susceptibility [22]. A recent study showed that the GG genotype of CTLA-4 CT 60 accounted for 36.2% of AIH patients, significantly higher than that of the control group (10.0%). The strength of the correlation between the two variables, as measured by Cramer’s *V*, was determined to be 0.341, indicating a moderate level of correlation. The bias-corrected Cramer’s *V* demonstrated a moderate correlation, exhibiting a correlation coefficient of 0.322 [23]. The CTLA-4 GG genotype significantly increases the risk of AIH-1 when patients have HLA DRB1*0301 in their genotype [22]. In Tunisian patients, the CTLA-4 + 49 position GG genotype was also positively associated with AIH susceptibility [24]. In addition, tumor necrosis factor-α-inducible protein 3 (TNFAIP3) is a genetically encoded ubiquitinating enzyme whose rs10499194 T allele and CT genotype are associated with an elevated risk of developing AIH. Thus, the rs10499194 polymorphism is an AIH locus for candidate susceptibility [25,26]. Patients in the Japanese population who had detrimental mutations in TNFAIP3 had an increased risk of cirrhosis-related AIH [27]. The receptor for Fas was found to be significantly elevated in lymphocytes from AIH patients [28]. Furthermore, the surface expression of Fas in CD4+ and CD8+ T lymphocyte subsets was significantly higher in AIH patients compared with normal subjects. Expanded Fas+ T cells also reflect the presence of persistent antigen-specific or non-specific activation in the body and abnormalities in the peripheral loss of activated lymphocytes [29]. A Japanese study observed that Fas polymorphisms and AIH development were strongly correlated with genetic factors. And the Fas-670A allele carriers showed an elevated risk of developing AIH [30]. Inflammatory cytokines are also involved in AIH development [31]. For instance, the IL-4-590 C/T polymorphism and the IL-4-33 TT genotype increase AIH susceptibility [32,33]. *In vivo* intervention with recombinant human interleukin-1 receptor antagonist (rhIL-1Ra) can reduce the secretion of TNF-alpha and interleukin-17 (IL-17) and the infiltration of inflammatory cells in the liver thereby inhibiting ConA-induced hepatitis [34]. CCN1 promotes inflammation by upregulating IL-6 production in ConA mice through the α6β1/PI3K/Akt/NF-κB pathway [35]. Recently, miRNAs have also demonstrated a crucial impact on AIH development [36,37,38]. A study comparing serum miRNAs showed significant differences between healthy individuals and AIH patients [39]. Compared with controls, serum levels of miR-29a were decreased, miR-378, let-7b, miR-122 and miR-192 were increased, and miR-574-3p, miR-193a-5p and miR-148a were unchanged. This indicates that the combination can be utilized to differentiate between people with AIH. Furthermore, a substantial correlation was found between miR-557 and a higher chance of recurrence. Additionally, it might also distinguish AIH patients from hepatitis C (HCV) patients, primary biliary hepatitis patients with cholangitis, and non-alcoholic steatohepatitis patients [40]. The expression level of miR-155 was significantly elevated in liver specimens of AIH patients but was reduced in peripheral blood mononuclear cells. In the CoA model, the level of miR-223 in Kupffer cells of mice with early liver damage was significantly increased, and transfection of miR-223 mimics could inhibit the activation of Kupffer cells after Con A stimulation [41].

### 2.2 Mechanisms of organismal immune imbalance

The liver is constantly exposed to various antigens due to its location and function, indicating the tolerance or responsiveness of the hepatic immune system [42]. Cytokines of immunosuppressive cells provide tolerance in the liver, migrating with immune mediators through the hepatic sinusoids to the liver parenchyma [43,44]. Under normal circumstances, the liver’s tolerance can result from the joint action of immune cells, parenchymal cells, epithelial and endothelial cells, and the microenvironment. The concentration of the immunosuppressive cytokine IL-10 is normally high in the liver. Besides, most liver antigen-presenting cells, including DC cells and liver sinusoidal endothelial cells, have low levels of MHC class II and co-stimulatory molecules CD80/CD86, thereby inducing an immune tolerance phenotype [15]. In addition, the proliferation of Foxp3+ Tregs in the liver mediated by the Notch signaling pathway can also release IL-10 and enhance the immunosuppressive environment [45]. Moreover, CD8+ T cells generated in lymph nodes have a strong intrahepatic antigen-specific immune response, while CD8+ T cells activated in the tolerogenic environment of the liver are more defective in inducing cytotoxic immune responses and have a short half-life [46]. During autoimmune diseases such as AIH, liver tolerance often collapses. Recent studies have revealed that the administration of anti-CD20 antibodies has the potential to considerably diminish the expression of MHC class II and CD80 molecules on B cells. Consequently, this intervention can decrease the capacity of B cells to deliver self-antigens to T cells, resulting in a reduction in T cell activation and proliferation. Additionally, this intervention alleviates inflammation in the liver and mitigates the breakdown of hepatocytes [47]. Chronic hepatitis is mainly caused by the interaction of multiple immune cells, such as lymphocytes, macrophages, natural killer T cells (NKT), etc. [48]. These cells secrete TNFα, interferon-gamma (IFN-γ), and other pro-inflammatory cytokines [49]. The liver inflammatory process could be initiated by presenting autoantigenic peptides to helper T-cell receptors, leading to Th1, Th2, and Th17 cell recruitment into the tissue [50]. These effector cells begin an immune response cascade depending on the release of cytokines. The regulatory T cells (Tregs) recruitment process involves lymphocytes interacting with surface molecules present in endothelial cells [51]. In the liver, Treg secretes the immunosuppressive cytokines IL-10 and transforming growth factor beta to inhibit the effector cell proliferation and action [52]. γδ T cells are innate lymphocytes recognizing non-peptide antigens and stress-induced ligands into two distinct subpopulations, including IFN-γ or IL-17-induced γδ T cell production [53]. Moreover, IL-17A, synthesized by γδ T cells, controlled hepatocyte injury in Jα18 knockout mice, an AIH model [54,55]. γδ T cells can also secrete IL-17 and cause damage to the liver [56]. Intraepithelial γδ lymphocytes are directly responsible for the cytolysis of effector cells and antigen-presenting cells through the granzyme-perforin, Fas-Fas ligand, and lymphotoxin pathways. They are a key population in the regulation of immune responses in tissues [57]. Both relative and absolute counts of γδ T cells were elevated in the peripheral blood of patients with AIH, which is characteristic of patients with AIH. Selective enrichment of the Vδ1 subpopulation of γδ T cells has also been found in Takayasu arteritis and systemic sclerosis, indicating that γδ T cells are involved in the pathogenesis of autoimmunity [58,59,60]. In addition, in another study, it was confirmed that the number of γδ T cells increased significantly during the active phase of AIH, and the production of its effector molecule granzyme B also increased significantly, which was consistent with the levels of liver damage markers alanine aminotransferase (ALT) and bilirubin. Consistent with the increase, the proportion of the Vδ1 subpopulation and the ability of this cell population to produce IFN-γ increased [61]. This process can promote B cells to stimulate plasma cells to release autoantibodies, causing damage to liver cells. In other autoimmune diseases, imbalances in the physiological levels of γδ T cells are also associated with inflammatory processes [59,60]. A recent study found that AIH models could also be generated by transferring γδ T cells from TOX-deficient mice into new mice [62]. It is well known that invariant natural killer T (iNKT) cells (also called type I NKT cells) express and characterize a semi-invariant T cell receptor (TCR). The TCR region of all iNKT cells contains the canonical Vα14-Jα18 TCRα chain. Thus, Jα18 knockout can hinder the synthesis of TCR and lead to the lack of NKT cells. In addition, the TCR of human and mouse NKT cells contains Vα14–Jα18 chain and Vα24–Jα18 chain, respectively. Therefore, Jα18 knockout mice have extremely important reference significance for the study of human Jα18 [63]. The current consensus among immune cells in AIH is that this autoimmune disease is caused by the signal presentation of self-antigens to uncommitted T helper (Th0) through HLA II molecules on antigen-presenting cells. At this time, Th0 cells are activated and differentiate into Th1, Th2, and Th17 cells according to different cytokines. When the cytokine environment is rich in IL-12, Th0 cells differentiate into Th1 cells. When the cytokine environment is rich in IL-4, Th0 cells differentiate into Th2 cells, while in the presence of IL-1β, IL-6, and TGF-β, Th0 cells differentiate into Th17 cells [64]. When differentiated into Th1 cells, these cells can secrete IL-2 and IFN-γ. The former cytokine can activate macrophages to secrete IL-1 and TNF-α, thereby damaging liver cells. The latter stimulates cytotoxic lymphocytes and enhances the expression of HLA class I molecules on APCs and HLA class II molecules on hepatocytes [65]. When differentiated into Th2 cells, IL-4, IL-10, and IL-13 can be produced, which can induce B cells to mature into plasma cells and then produce autoantibodies [20]. Finally, when the environment is rich in IL-6 and TGF-β, these cytokines will inhibit the function of Treg cells and induce the differentiation of Th0 cells into Th2 cells [64]. In addition, NKT cells have been shown to be significantly reduced in AIH patients, which leads to reduced secretion of IL-4, an inhibitor of Th17 development [66]. Th17 will secrete IL-17, IL-22, and TNFα, causing damage to liver cells and inducing hepatocytes to secrete IL-6. The secreted IL-6 will further stimulate Th17 cells to produce corresponding cytokines [67]. During this process, γδ T cells can also secrete IL-17 to play a similar role [56]. Humoral immunity is also associated with liver injury in AIH [68]. As we know, anti-nuclear autoantibodies (ANA) and/or anti-smooth muscle autoantibodies (SMA) are positive in AIH-1, while anti-liver-kidney microsomal and/or anti-hepatic cytoplasmic antibodies are present in AIH-2. The autoantigens targeted by AIH-2 autoantibodies are anti-liver and kidney microsomal antibody type 1 (LKM1) cytochrome P450 2D6, CYP2D6, and anti-lc1 formyl aminotransferase-ring deaminase. Stimulation of CYP2D6 favors Th1 responses as its peptide aa 305–324 can induce high levels of interferon production [20]. In models of AIH caused by CYP2D6 plasmids, there was a persistent elevation in transaminases, chronic inflammation, and liver fibrosis. These changes were accompanied by increased Th1 responses and decreased liver-infiltrating Treg cells [69]. In addition, CYP2D6 can be inhibited by LKM1-positive serum *in vitro*, leading to hepatocyte damage, which was mentioned in the manuscript. LKM1 could be a diagnostic marker for AIH type 2. Cytochrome P4502D6 (CYP2D6) protein can be found on the surface of human liver cells. This cytochrome can be inhibited by LKM1-positive serum *in vitro*, leading to hepatocyte damage [5].

### 2.3 Non-immunomodulatory factors

Autoimmune diseases exhibit heterogeneity, manifesting as the involvement of specific or several organ systems. A fundamental association exists between environmental exposures and the likelihood of experiencing adverse health outcomes, wherein the influence on the immune system surpasses genetic abnormalities [70]. Studies have shown that gender is one of the influencing factors for the occurrence of AIH. Whether it is type 1 or type 2 AIH, women’s incidence rate is higher than men’s [71]. 40% of type 1 and 80% of type 2 AIH cases occur before the age of 18 years [72,73]. Postmenopausal women are also in high-risk groups [74]. When the iodine content in the environment is high, the overall incidence of AIH is significantly higher than that in areas with low iodine content [75]. Viruses with hepatophilia significantly induce autoimmunity due to inflammatory responses in the local regions. The generated cellular immune response can eradicate the pathogen. For instance, anti-LKM1 antibodies have been identified in HCV against CYP2D6 [5]. A study from Brazil assessed AIH frequency in chronic HCV patients. Epidemiologically, HCV infection is correlated with AIH and is also a triggering factor [76]. SLA/LP shares high sequence similarity with a non-homologous Rickettsiae protein, driving autoimmune responses through CD4+ T cell autoantigen recognition and concomitant humoral immunity [77]. A New Zealand-based study assessed the impact of environmental factors on the risk associated with AIH. Moreover, the univariate analysis of the study revealed that alcohol consumption reduced AIH risk [78]. Lifestyle factors, childhood factors, and family history of 72 AIH cases and 144 controls, who lived in Canterbury, New Zealand, between July 1, 2011 and June 30, 2012, were collected. Analysis performed by comparing AIH with drinking concluded that alcohol consumption was independently associated with a reduced risk of developing AIH, with OR 0.43, 95% CI 0.28–0.68, *p* < 0.01 [78]. In another study, it can also be found that the alcohol consumption of AIH patients is lower than that of alcoholic liver disease (ALD) patients and AIH/ALD patients [79]. Upon evaluating the correlation between vitamin D levels and histological characteristics and therapy response in patients with AIH, it was shown that the AIH group exhibited notably lower levels of vitamin D compared to the control group. Furthermore, a strong correlation was observed between interfacial hepatitis and fibrosis scores and low levels of vitamin D. It was shown that patients with these characteristics exhibited inadequate responses to treatment [80]. Intestinal microbial ecology is critical to developing intestinal and systemic immune responses [81]. The gut microbiota could be a rich antigen reservoir, initiating and maintaining the autoimmune reaction to AIH. Due to its unique anatomical location, the liver is closely associated with the gut microbiota, playing a significant role in the development of AIH. Recent research has indicated an observed modification in the gut microbiome of individuals with AIH and primary biliary cholangitis. This finding suggests a potential association between the alteration of gut microbiomes and the development of AIH [82]. Liu et al. found that treating AIH model mice with Bifidobacterium and Lactobacillus can significantly increase Treg cell infiltration, inhibit the production of Th1 and Th17 cells and inflammatory factors, and inhibit the activation of Toll-like receptor 4 (TLR4)/NF-κB pathway to alleviate the progression of AIH [83]. The role of gut microbiota in humans has also been studied accordingly. Liwinski et al. found that compared with control group, biodiversity in the gut microbiota of AIH patients was reduced. Consistent with the results obtained in the AIH mouse model, the abundance of Bifidobacterium flora was also significantly reduced in AIH patients and affected the AIH progression [84]. The abundance of Bifidobacteria, Lactobacillus, Bacteroides, and *C. leptum* in the gut microbiota of AIH patients was also significantly reduced, while the abundance of *Escherichia coli* was increased [84]. After FMT treatment in the Abx experimental autoimmune hepatitis (EAH) mouse model, the liver injury and bacterial translocation of the mice were reversed to varying degrees [85]. Thus, molecular mimicry can describe the process behind inducing autoimmunity through microbes [86]. Compared to normal structures in healthy controls, one study indicated altered intestinal tight junctions and inflammatory infiltrates within the lamina propria of AIH cases [87].

The drug-induced acute liver failure manifestations could be similar to AIH. Drugs could induce AIH through genetic susceptibility and molecular mimetic mechanisms, depicting the etiology of drug-induced liver injury (DILI) associated with autoimmune features [88]. Minocycline and furantoin are associated with AIH among the studied drugs and compounds [89]. The drug-induced AIH mechanisms include drug metabolite production that binds to receptors and acts as antigenic complexes. These stimulate autoantibody production, primarily CYP1A2 and CYP2A6, and lymphocyte sensitization [90]. The tissue and interaction feature of AIH characterize DILI with autoimmune features. In contrast to medication-induced AIH, autoimmune DILI manifests symptoms of liver damage and jaundice during a brief incubation time. A liver biopsy reveals inflammation within an infiltrate primarily composed of lymphocytes and eosinophils. Following withdrawal, there is a gradual recovery with a danger of relapse from re-administration [91].

## 3 Histopathology

AIH pathology is diverse and dependents on inflammatory responses of hepatic parenchymal cells due to an autoimmune imbalance [92]. The characteristic clinical manifestations include portal and periportal lymphoplasmacytic infiltration and interfacial hepatitis. Thus, single or clusters of plasma cells could be observed in the lobules [93]. However, nearly 1/3 of AIH patients have scarce or absent plasma cells [94]. AIH commonly involves ballooning degeneration, punctate hepatocyte necrosis, and apoptotic vesicles. Moreover, the inflammation and necrosis severity varies from mild and active hepatitis to bridging even extensive necrosis [95]. The regenerating hepatocytes had a rosette-like structure (2–3 aqueous degenerated hepatocytes developing a pseudo glandular system possessing dilated capillaries in the center, depicting a rosette) [96]. The reticular fibrous scaffold collapses in the necrotic area due to further progression. Thus, hepatic stellate cell activation and pseudobullet formation lead to hepatic fibrosis [97]. Around 65% of patients suffer penetration (lymphocyte entry into hepatocytes), leading to apoptosis, and its appearance correlates with inflammation degree and fibrosis in the liver [98]. In some AIH cases, central lobular injury with significant hepatocyte necrosis and mononuclear inflammation could be related to periportal activity and interfacial hepatitis [99]. Thus, chronic hepatitis injury could represent an early disease stage because the central lobular necrosis injury pattern can become a more typical portal-based form [100]. Nevertheless, this pattern of necroinflammatory injury to the central lobules is not specific to AIH; instead, it is more frequently encountered in hepatitis caused by viruses or drugs [101]. Bile duct disruption is not observed in AIH. However, up to 12% of biopsies indicate bile duct disruption; another 12% demonstrate lymphocytes infiltrating the bile duct epithelium [102].

## 4 Clinical manifestations and diagnostic criteria

AIH has an insidious onset and a variety of clinical manifestations, mostly with chronic non-specific symptoms, such as malaise, fatigue, general malaise, etc. Signs such as hepatosplenomegaly and ascites may be present [103]. Patients with AIH without obvious clinical symptoms account for 25%. About 1/3 of patients have progressed to the stage of liver fibrosis or cirrhosis by diagnosis [104]. The presentation of acute AIH may be similar to the symptoms of acute hepatitis due to other causes. It may present with elevated ALT, aspartate aminotransferase (AST), and jaundice [105]. Patients with AIH may also present with symptoms of intrahepatic cholestasis, and such patients may present with a failure to respond to glucocorticoid therapy. About 10% of patients are negative for autoantibodies [106]. These patients often show mildly elevated or normal IgG levels and rely on histological features for diagnosis. AIH is often combined with other autoimmune diseases, such as inflammatory bowel disease and Hashimoto’s thyroiditis. Although we can diagnose AIH through the above diagnostic criteria, we still need to pay attention to the differential diagnosis of some diseases during implementation [107]. First, we must rule out acute and chronic viral hepatitis caused by viruses (such as HAV, HBV, EBV, or CMV). The syndromes of primary biliary cirrhosis and primary sclerosing cholangitis partially overlap with AIH. These can all be distinguished by testing blood samples for the corresponding antibodies [108]. Second, Wilson’s disease is a differential disease that cannot be bypassed. It is biochemically, serologically, and histologically similar to AIH, but it can be initially identified by measuring liver copper concentration and 24 h urinary copper excretion [109,110]. Leishmaniasis and other hepatic infectious diseases may also need to be excluded based on local factors or travel history [111]. Furthermore, a novel approach that has surfaced recently involves using the exchangeable copper assay to determine the presence of copper overload and potentially differentiate individuals with Wilson’s disease [112]. KF ring is a classic ophthalmic manifestation of Wilson’s disease and can be examined by slit lamp. The clinical manifestations of DILI are similar to AIH and it is quite difficult to identify these diseases. There are currently several methods available for differential diagnosis. First, it is necessary to exclude medication history. Additionally, the utilization of the RUCAM scale and M&V scale is employed to ascertain the causal association between pharmaceutical substances and hepatic injury. Ultimately, the acquisition of histological specimens via liver biopsy serves as a means to achieve a pathological diagnosis [113,114]. In addition, non-alcoholic fatty liver disease is also a disease that needs to be differentially diagnosed with AIH. The two are mainly distinguished through the NASH and liver tissue biopsy diagnostic criteria [115].

AIH diagnosis is based on clinical, biochemical, serological, and histological features [116]. The diagnostic criteria for AIH were established and revised by the international autoimmune hepatitis group (IAIHG), and the system grades the clinical, laboratory, and histological features of AIH [117]. Initially, it was designed to standardize AIH diagnosis, but later, it was applied in a clinical setting. The IAIHG scoring system involves the difference between a definite and probable AIH diagnosis, primarily associated with the increased serum gamma globulin or IgG, ANA, ASMA, or anti-LKM-1 levels, and exposure to alcohol, drugs, or infections causing liver damage [118]. Treatment response improves AIH diagnosis and is a part of the scoring system. As is shown in Table 1, there are differences in the scoring categories before and after treatment. Before treatment, clinical characteristics, laboratory tests, and liver histopathology need to be combined to score. If these scores are higher than 16 points, the diagnosis can be confirmed, and 10–15 points indicate the possibility of AIH. If the score is less than 10 points, the diagnosis of AIH can be ruled out. After treatment, the patient’s response to treatment must also be considered for scoring in addition to the above items. If the score is higher than 18 points after treatment, the diagnosis is definite, and 12–17 points is a possible diagnosis [119].

**Table 1:** IAIHG revised diagnostic scoring system

Parameters/clinical characteristics	Scoring	Parameters/clinical characteristics	Scoring
Female	+2	Histological examination of the liver	
Ratio of ALP to AST (or ALT)	+2	Interface hepatitis	+3
<1.5	0	Mainly lymphoplasmacytic infiltration	+1
1.5–3.0	−2		
>3.0		Hepatocytes show rosette-like changes	+1
ANA, ASMA, or anti-LKM-1 antibody titers	+3	No such manifestations	−5
>1:80	+2	Bile duct changes	−3
1:80	+1	Other changes	−3
1:40	0	Other immune diseases	+2
<1:40	−4	Other available parameters	
AMA positive		Positive for other specific autoantibodies (anti-SLA/LP antibody, anti-LC-1 antibody, ASGPR.pANCA)	+2
Hepatitis virus markers	−3		
Positive	+3		
Negative			
Drug history	−4	HLA-DR3 or DR4	+1
Positive	+1	Response to treatment	
Negative		Complete	+2
Average ethanol intake (g/day)	+2	Relapse	+3
<25	−2		
>60			
Explanation of total points			
Before treatment		After treatment	
Definite AIH	≥16	Definite AIH	≥18
Possible AIH	10–15	Probable AIH	12–17

ALP, alkaline phosphatase; AST, aspartate aminotransferase; ALT, alanine aminotransferase; IgG, immunoglobulin G; ANA, antinuclear antibody; SMA, anti-smooth muscle antibody; anti-LKM-1 antibody, anti-liver and kidney microsomal antibody type I; AMA, anti-mitochondrial antibody; anti-LC-1 antibody, anti-hepatocyte cytoplasmic antibody type 1; ASGPR, anti-desialytic acid Glycoprotein receptor antibody; pANCA, perinuclear type anti-neutrophil cytoplasmic antibody; HLA, human leukocyte antigen.

Although the diagnostic criteria of AIH are relatively clear, its diagnosis is still challenging. To some extent, AIH is still a disease of exclusion, which needs to be differentiated from many similar diseases. In the case of acute hepatitis after infection with acute autochthonous hepatitis E virus, it behaves similar to AIH [120]. In addition, Wilson’s disease, Leishmaniasis, and other infectious diseases of the liver also need to be excluded. Due to differences in the preparation process, the diagnostic antibodies for AIH cannot be consistent in terms of false positives and false negatives. The expression of AIH-specific antigens has been observed in animals’ kidneys, livers, and stomachs. This poses significant challenges in establishing a unified standard for immunofluorescence detection [121]. Although anti-SLA antibody has high specificity in patients, it is still powerless in the face of the AIH subgroup and SLA positive but negative for other antibodies [122]. Moreover, corresponding antibodies only emerge during further disease progression and may be absent in acute AIH. Therefore, retesting for antibodies is encouraged. On the other hand, autoantibodies (ANA and SMA) are frequently detected in almost any type of acute severe hepatitis, including DILI [123]. There remain several pressing diagnostic issues that require immediate resolution. A more precise diagnosis can also offer valuable insights for clinical treatment, enabling patients to achieve enhanced therapeutic outcomes.

## 5 Treatment

Successful treatment can reduce AIH inflammation and prevent progressive fibrosis while minimizing the therapeutic side effects [124]. The current standard treatment for AIH is corticosteroids with or without azathioprine (AZA) [125,126]. Most patients respond well to this standard treatment. Only a minority of patients need alternative treatment modalities because they cannot tolerate the regimen or withdraw therapy due to side effects. Although corticosteroids are very effective in inducing remission, the usage must be tapered gradually and eventually discontinued after about 12 months due to side effects [127]. Budesonide has the potential to serve both as a primary and secondary therapeutic agent in the induction of mild early AIH. The efficacy and safety of MMF and tacrolimus as second-line agents have been extensively investigated. However, the available data consist of minimal retrospective trials with varying characteristics [128].

Since the 1950s and 1960s, prednisone combined with AZA has been the standard of care [129]. Most patients respond well to standard therapy; others are intolerant or non-responders and require alternative regimens. Around 10–15% of patients discontinued standard immunosuppressive treatment due to intolerable side effects, which became 18% in jaundiced AIH patients [130]. The relapse rate after discontinuing standard immunosuppressive therapy was >50%. Prednisone can effectively induce remission and, under ideal circumstances, should be tapered and used after 12 months. However, a UK analysis indicated that 55% of patients still require hormone maintenance therapy [131]. Current maintenance therapy with long-term low-dose steroid therapy maintains the immunosuppressive effect, minimizing the probability of relapse [132]. The long-term low-dose steroid medication administration in maintenance therapy effectively sustains the immunosuppressive impact, reducing the likelihood of recurrence. Meanwhile, the extended use of hormones harms patients’ overall well-being with AIH, regardless of the specific hormone used, dosage, or the patient’s biochemical remission status [133]. Prednisone and AZA have significant side effects during treatment. Corticosteroids can significantly reduce the patient’s activity ability and increase anxiety, depression, and fatigue [134]. Prednisone can also induce hepatic insulin resistance in AIH patients and increase the risk of diabetes, with about 6–20% of new diabetic patients. In addition, patients may also face side effects such as hypertension, moon face, acne, cataract, psychosis, and low trauma fracture [131]. In addition, previous studies have shown that when corticosteroids are completely discontinued from medication in AIH patients, the average body weight per patient can be reduced by 6 kg [135]. Besides, AZA can induce myelosuppression, hepatotoxicity, and pancreatitis, which are at higher risk in patients with cirrhosis [136]. Withdrawal can be caused by a variety of adverse events. Approximately 13% of patients withdrew prematurely due to treatment-related side effects. These adverse effects included intolerable body shape changes or obesity (47%), osteoporosis with spinal compression (27%), brittle diabetes (20%), and peptic ulcer (6%) [137]. Although AZA can be used with corticosteroids, AZA must be discontinued if cholestatic hepatitis, pancreatitis, progressive leukopenia, or gastrointestinal discomfort occurs during the medication [137].

Budesonide has a 90% first-pass hepatic clearance, showing a more anti-infective effect than prednisone, with minimal adverse effects [138]. A prospective, double-blind, randomized trial observed that budesonide combined with AZA was superior to prednisone and AZA in treating non-cirrhotic AIH patients. However, there was a significantly lower incidence of steroid-specific adverse reactions. In contrast, emerging case reports after the trial indicated that AIH reactivation occurred during budesonide monotherapy [139]. A German retrospective study observed a biochemical response in 70% of patients after switching monotherapy from prednisolone to budesonide after 12 months. Later, a biochemical response was observed in 67% of patients after 24 months [140]. During the last follow-up (mean 63 months), 25% of patients were switched to prednisolone due to a poor budesonide response or related side effects.

Mycophenolate mofetil (MMF) inhibits purine production necessary for the ab initio lymphocyte synthesis during proliferation, thereby reducing inflammatory cytokines and elevating Treg cells. However, MMF has low systemic bioavailability, is teratogenic, and is not advised during pregnancy [141,142]. Saitis et al. reported that 88% of patients achieved biochemical remission after the combined treatment of MMF with prednisone for 26 months in 59 primary AIH patients [143]. Another study treated 109 patients with a combination of prednisolone and MMF and compared them with 22 patients treated with a combination of prednisolone and AZA. Around 72% of MMF group patients showed complete remission, which had a higher remission probability than the AZA group. In various small retrospective studies, the biochemical remission rates of MMF ranged between 31 and 73%, while the discontinuation rates ranged between 13 and 34% [144]. MMF is a second-line treatment option for non-responders or those intolerant to AZA.

Cyclosporine and tacrolimus inhibit calcium-regulated neurophosphatase, resisting T-cell activation and cytokine production [145]. Children with AIH exhibit good biochemical responses with cyclosporine for 6 months. However, the efficacy and safety of cyclosporine in children have no long-term evaluation. Cyclosporine has side effects such as nephrotoxicity and neurotoxicity [146]. Tacrolimus efficacy in AIH treatment in adults has been reported differently. Combining the three studies, the biochemical response rate for patients aged 60–62 years who failed tacrolimus treatment was 92%. In contrast, only 27% achieved a complete biochemical response in a recent report on 17 adults and 6 children [147]. Another study assessed the efficacy of MMF and tacrolimus as second-line treatment. The complete remission rate (69.4%) in the MMF group was not significantly different from the tacrolimus group (72.5%). Both drugs were equally effective in patients where standard therapy failed. Tacrolimus showed a higher complete remission rate than MMF (56.5% vs 34.0%) [148]. Therefore, tacrolimus could be a second-line agent for AZA intolerance or poor response in AIH patients, particularly those unable to use MMF [99]. Treg cells are essential in humans as loss of function leads to autoimmunity [149]. mTOR can regulate several critical cellular processes, including nutrient sensing, cell proliferation, and metabolism. mTOR controls cytokine production and T-cell differentiation in T cells. Abrogated mTORC1 and mTORC2 activities can reduce the differentiation of Th1, Th2, and Th17 and spontaneously induce Treg cells, while single abrogated mTORC1 activity will increase the differentiation of Th2 cells [150,151]. Rapamycin (mTOR) inhibitors restrict effector T cell differentiation and enhance Treg proliferation [152]. Moreover, Treg cells treated with rapamycin decreased the secretion of IL-2 and IL17, as well as the levels of glycolysis and oxidative phosphorylation [152]. In addition, mTOR signaling can regulate the stage-specific development of T cells. Lack of RAPTOR at the DN1/DN2 stage decreased T cell proliferation and increased apoptosis [153]. mTORC2 activity in T cells is also necessary to induce glycolysis and Notch-driven proliferation. Also, it has significant regulatory effects on the expression of receptors involved in thymocyte development [154,155]. Notably, AKT blocked the generation of tTreg cells in the thymus by increasing mTORC1 activity [156]. TSC1 deficiency can promote tTreg cell development by inhibiting mTORC2-dependent signaling pathways [157]. Kerkar reported six patients with failed AZA/MMF therapy after liver transplantation who were treated with sirolimus. The median biochemical response time was 4.6 months, with one patient discontinuing treatment due to infection [158]. Several therapeutic pathways are being developed, such as interventions targeting gut microbial alterations and bacterial translocation, B-cell regulation, Treg cell transplantation, and miRNAs [159]. For instance, comparing 24 AIH patients and 8 healthy volunteers showed that the patients had dysbiosis, impaired intestinal barrier function, and elevated bacterial translocation (increased plasma LPS levels) related to disease severity [160]. LPS activates TLR4 in Kupffer cells, triggering an inflammatory response. LPS also activates TLR4 in hepatic stellate cells, enhancing the secretion of chemokines and adhesion molecules by Kupffer cells with elevated chemotactic ability [161]. JKB-122 is an antagonist of TLR4 with hepatoprotective and anti-inflammatory activity. JKB-122 can reduce levels of pro-inflammatory cytokines in the serum and liver of an AIH animal. JKB-122 has a dose-dependent biochemical and histological improvement in AIH, and is currently in Phase II clinical trials [162].

## 6 Animal models of AIH

Although there are many treatment options currently available, each of these treatment options has shortcomings that need to be overcome. Therefore, animal models are a better choice for drug development. Besides, it can provide considerable significance for the study of the mechanism of AIH and provide an early basis for further preclinical trials.

Animal AIH models are primarily divided into spontaneous (naturally occurring or knockout) and induced (biological or physicochemical factors). They provide information about hepatic immunology or cause liver damage mediated by the immune system. Due to their close connection with AIH, these models aid in evaluating novel therapeutic strategies for human diseases. The ideal experimental model should resemble the human condition closely. However, mice mimicking human autoimmune disorders have different autoantigen targets. This is due to class I and II molecule differences in MHC, with specific T-cell responses distinguishing mice from humans [163]. The animal models employed in the study of AIH are briefly described and shown in Table 2.

**Table 2:** Mouse model of AIH

Animal model	Animal	Applications	Advantages	Disadvantages
Immunization with allogeneic or xenogeneic liver proteins	Mouse	Basic mechanisms of infiltration of AIH	Produced anti-LSP antibodies	Difficult to differentiate from other diseases
			Easy preparation of disease model	It is challenging to study the role of immune factors in AIH
			Elucidated the relationship between genetic background and disease susceptibility	Lack of pathogenic T cells
				Induction of mild or moderate hepatitis in the liver
Immunization with syngeneic liver homogenate or liver-specific lipoproteins	SMA mouse	Basic mechanisms of infiltrations autoantibodies of AIH	Portal infiltration of mononuclear cells in the liver and necrosis of hepatic parenchymal cells	Immune responses to AIH-unrelated antigens
			Susceptible to hepatitis	It is difficult to simulate the process of chronic or mild hepatitis
			Production of antibodies and corresponding cytokines	
Immunization with syngeneic liver homogenate	C57BL/6 mouse or A/J mouse	Basic mechanisms of antibodies and T cells in AIH	Presence of immunosuppressive T cells	Autoantigen complex, autoantigens with unknown components
			Easy preparation of disease model	
			No transgenic animals involved	It is challenging to simulate chronic hepatitis
			No complicated methods of inducing disease	
Immunization with ConA	Mouse	Basic mechanisms of severe acute hepatitis and T cell activation	Exploration of antigen-independent cytokine storm-mediated liver injury	No chronic changes
			Discovery of drug treatment options for liver damage	
Genetically modified spontaneous or induced immunization	Mouse	Role of immune response and tolerance	Elucidated the link between genetic background and specific genes	Single genes have limitations in mediating AIH
			Identified the role of single genes in immune response/tolerance	AIH usually involves multiple organs
			Exploration of the association between influencing factors and single genes	

The first animal AIH models were developed in the early 1970s when more fundamental immune system mechanisms were discovered. During that time, AIH had a transparent immune-mediated background. Büschenfelde et al. injected liverspecific antigens LP-1 and LP-2 fractions from human liver homogenates into rabbits and various adjuvants. They observed that the large LDL LP-1 effectively induced “experimental immune hepatitis” [164]. LP-1 is a liver-specific membrane lipoprotein (LSP), identified as ASGPR. LP-1 is highly expressed on the hepatocyte surface. Anti-LSP antibodies could be observed in nearly 88% of AIH patients and can become a general marker. However, these antibodies are not for diagnostic purposes, as they are also detected in chronic hepatitis B and C, ALD, and primary sclerosing cholangitis patients [165]. Besides, Sundin et al. found that the incidence of anti-LSP antibodies detectable in the serum of nearly 55% of chronic active hepatitis patients was found by ELISA, compared with 17% in healthy controls, so it can be concluded that some healthy people also have anti-LSP antibodies [166]. The experimental rabbits synthesized antibodies against LP-1 and LP-2. However, AIH, similar to chronic hepatitis, was not triggered, demanding cellular immunity activation. In 1983, Kuriki injected homozygous liver homogenate or liver-specific lipoprotein with *Klebsiella pneumoniae* 03:K1 lipopolysaccharide as an adjuvant inside SMA mice. This led to anti hepatocyte-specific lipoprotein antibody production and monocyte infiltration in the liver [167]. The model indicated pathogenic autoimmune cells, as spleen cells were transferred to other mice after 14 days. Characteristics including liver parenchymal cell necrosis and monocyte portal infiltration were noted, which were also present in the donor mice. To create the intricate EAH model, Lohse administered homogenous liver homogenate (S100) supernatant into C57BL/6 mice, which had been emulsified with Freund’s adjuvant [168]. S100 extracts had several liver autoantigens, including CYP2D6 and SLA/LP. The EAH model induces the production of S100 protein-specific T cells and transient liver injury (perivascular infiltration and hepatocyte necrosis). Consequently, this study provides significant insights into the identification and control mechanisms of effector cells involved in immune responses specific to the liver. Its simplicity characterizes the S100 model, as it does not require transgenic mice or intricate techniques for disease induction. Nevertheless, inducing comparable chronic recurrent AIH is a significant challenge in these initial animals. Therefore, a comprehensive understanding of target antigens is necessary to track the action mechanism of hepatic autoantigen-specific T lymphocytes.

**Figure 1:**
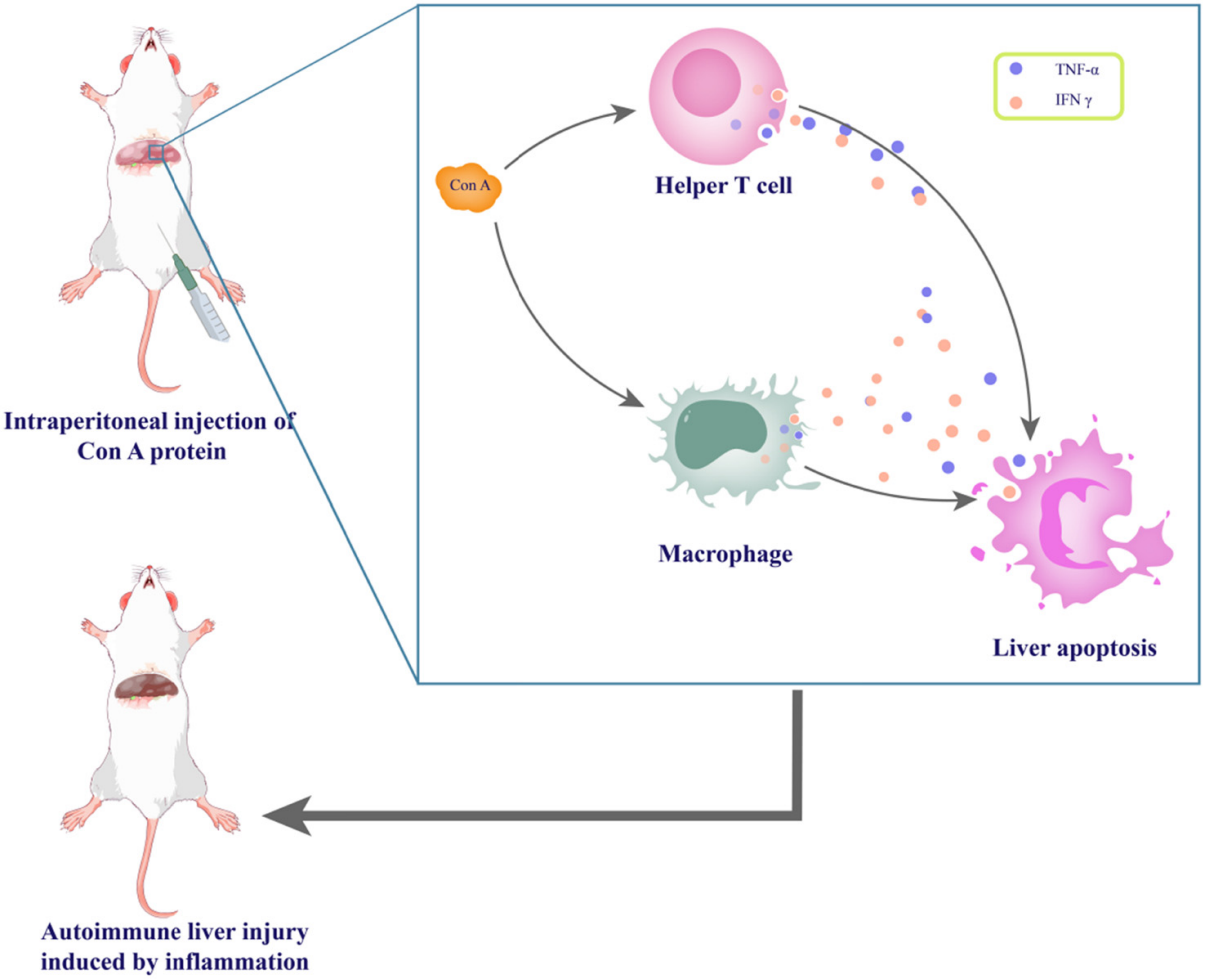
Schematic illustration of the main mechanisms of acute immune liver injury induced by ConA.

Since the early 1990s, systemic application of Ladin A (ConA) was frequently used to induce liver injury in mice. This activated non-specific T-cell antigens caused acute hepatitis. ConA, a phytohemagglutinin, is a potent mitogen causing cytokine storms. It amplifies through lymphocyte and macrophage interactions, causing inflammation, apoptosis, and liver failure [169]. Further studies indicated that the acute liver injury model in ConA-induced EAH mice primarily interacts through Th1 cytokines. Thus, NKT cells are also essential in liver injury caused by ConA [170]. The ConA hepatitis model is reliable for studying immune system-mediated liver injury. Nevertheless, this model demonstrates a more severe form of liver injury characterized by a non-dependent cytokine storm triggered by hepatic autoantigens, as opposed to long-term changes (Figure 1).

ConA is a widely used mitogen that activates T cells and depends on helper T lymphocytes (Th) and macrophages for its effects. ConA activates T lymphocytes and stimulates Th cells and macrophages to produce cytokines such as TNF-α and IFNγ. TNF-α can directly damage hepatocytes and lead to apoptosis. After intravenous injection of ConA into mice, most of it accumulates in the liver, indicating that it is the target organ for ConA toxicity induction *in vivo*. In the inflammatory process of viral hepatitis, lymphocyte-mediated cytotoxicity is the primary immune mechanism leading to the lysis of virus-infected hepatic parenchymal cells. The adhesion between lymphocytes, antigen-presenting cells, and antigen-producing target cells is essential for the immune response process. The experimental animal model of ConA-induced liver injury in mice is pathologically characterized mainly by activation of T lymphocytes resulting in Acute immune liver injury.

The transgenic mice emergence overexpresses specific proteins or lack one or more proteins under system- or organ-specific promoters (KO mice). Many new AIH models have been developed, demonstrating new findings associated with AIH immunopathology. One model is the NTxPD-1−/− mice, where the programmed cell death 1 (PD-1) gene is absent and excised from the thymus [171]. NTxPD-1−/− mice do not possess adequate regulatory T cells to facilitate normal homeostasis, similar to normal PD-1-deficient mice. The thymus gland removal leads to AIH-like hepatitis development. This leads to T-cells entering the liver parenchyma, liver lobule necrosis, and ANA production. CXCL9 blockade inhibits the lethal AIH progression in NTxPD-1−/− mice by reducing the CXCR3+ T cell production. Therefore, CXCL9 chemokines coordinate the movement of T-cells into the liver from the spleen. The effect of T cell priming depends on the overall antigen load. When antigen load is high, T cell priming leads to the exhaustion of T cells due to the abundant surface expression of programmed cell death protein 1 (PD-1) [172]. Loss of both Tregs and PD-1-mediated signaling leads to lethal AIH [171]. The increased expression of PD-L1 and B7-DC on Kupffer cells and hepatic sinusoidal endothelial cells can bind to PD-1 on T cells, suppressing autoreactive lymphocytes, which regulates the progression of AIH [173]. Furthermore, loss of PD-1 resulted in enhanced proliferative capacity of T cells in the viral liver [174]. PD-1 can also regulate the migration ability of T cells. Blockade of chemokine ligand 20 inhibits the migration of splenic T cells expressing chemokine receptor 6 to the liver and suppresses the development of AIH in the absence of PD-1 [175]. Furthermore, anti-PD-1 antibodies in serum help differentiate AIH-1 from DILI [176]. Since mice mutations led to multi-organ inflammatory damage, the vital role of transforming growth factor receptor-1 (TGF-β1) in the homeostasis of immune cells is described. Balb/c TGF-β1−/− mice are susceptible to spontaneous and extensive inflammation in the liver and hepatocyte necrosis, with increased levels of plasma ALT [177]. Balb/c TGF-β1−/− mice inflammation and necrosis were attributed to CD4+ T cells and the secreted regulation of IFNγ-mediated immunity, similar to humans. The AIH model was constructed with autoantigens recognized by antibodies or T cell targets in AIH patients to mimic the autoimmune response and the ability to activate autoantigen-specific of immune cells in human AIH. Besides, TGF-β can promote the differentiation of Th0 cells into Treg cells in the immune microenvironment. In addition, under the joint action of TGF-b, IL-1, and IL-6, the differentiation of Th0 cells into Th17 cells is enhanced [15]. Previous experiments have confirmed that TGF-β is also an important mediator of AIH. Serum levels of TGF-β were significantly elevated in mild AIH patients [178]. PD-1 and TGF-β can synergistically promote the development of Treg cells. The joint action of PD-1 and TGF-β can inhibit the phosphorylation of Smad3 mediated by cdk2, and enhance the deactivation of Smad3 in a TGF-β-independent manner to promote the differentiation of naive T cells into Treg cells [179]. CYP2D6 is the characteristic antigen identified using T cells and antibodies in AIH-2 patients. The CYP2D6 mice model uses the same antigen in humans as the trigger. This antigen is injected into the wild-type C57BL/6 mice liver by infecting an adenovirus with the encoded human gene. The triggered self-responses target mouse CYP2D6 homologs since mice lack human CYP2D6 [180]. Adenovirus-infected mice cause acute hepatitis, which causes chronic AIH resembling interfacial hepatitis. There is inflammatory cell infiltration, such as CD4+ and CD8+ T cells, B cells, macrophages, dendritic cells, and neutrophils, in interfacial hepatitis. Moreover, mice synthesize autoantibodies similar to LKM-1, primarily recognizing CYP2D6 epitopes similar to AIH-2 patients. T-cell epitope mapping identified one CD4+ and three distinct CD8+ T-cell epitopes, not corresponding to AIH patients due to MHC molecule differences [18]. However, the T cell epitope is in the crossover region between human CYP2D6 and murine CYP homologs. Thus, AIH pathogenesis could be associated with molecular mimicry. The CYP2D6 model can elucidate the persistent fibrosis mechanism related to AIH. Hepatic stellate cells are activated after the adenovirus infection. This increases the extracellular matrix component expression, such as the deposition of type I and type III collagen and alpha-smooth muscle actin [181]. Gil-Farina et al. injected adeno-associated viruses with IL-12 gene overexpression into mice and observed type I AIH characteristics [182]. However, none of the above models appropriately depicts the human AIH. Some models are better adapted to understanding immune activation or regulatory mechanisms, while others aid in evaluating innovative therapeutic methods.

## 7 Future therapy strategies

Most current studies have recognized the role of immune response and tolerance in AIH. Hence, it is imperative to select pharmaceutical agents that exhibit minimal adverse effects on prolonged immunosuppression in the management of AIH. Additionally, exploring potential alternative therapies in cases where the initial treatment fails to provide a satisfactory response is crucial. As previously stated, tacrolimus demonstrates notable efficacy in treating patients with AIH. It may be an alternative therapeutic option for those who exhibit intolerance or experience adverse events to AZA, particularly those who cannot tolerate MMF. However, its current side effects still need to be overcome. Liver-specific autoantigen epitopes recognized by TCRs have not been identified in AIH-1. Autoantibodies and clear autoantigen epitopes of TCRs also enable new options for transgenic animal models and treatments in type 2 AIH. Thus, mechanistic exploration is still essential for AIH-1. Future treatment can start from several aspects based on the current research progress. One area of focus is the advancement of novel immunosuppressants that exhibit enhanced specificity for AIH, thereby effectively managing hepatic damage.

Additionally, there is a need to focus on advancing pharmaceutical interventions to reinstate immune regulatory mechanisms and promote antigen-specific tolerance. Furthermore, regulating autoreactive CD4 and CD8 T cell proliferation and elimination is crucial. In conclusion, there is a pressing need to enhance the efficacy of anti-fibrotic medications to mitigate the onset of liver fibrosis or maybe reverse the existing condition. These elements are anticipated to serve as novel therapeutic avenues.

## 8 Conclusion and outlook

Early diagnosis and treatment of AIH can facilitate good health and reduce the societal burden of chronic diseases. However, there are many unanswered questions in studying AIH mechanisms. These include racial differences, genetic heterogeneity, social environment, and other disease development factors. Therefore, large samples, multicentric and cross-ethnic studies, and developing animal models similar to human AIH can further elucidate AIH pathogenesis. Disease awareness and timely diagnosis are crucial, as untreated disease has a poor prognosis. AIH diagnosis is exclusionary based on various combinations. Diagnosis includes laboratory tests and diagnostic scores, but most are not disease-specific markers, such as autoantibodies, elevated IgG/gamma globulin levels, histological features, and exclusion of other liver diseases. Thus, supplementing diagnostic-specific markers and gaps in high-risk predictors is the requirement. There is still room for improvement in the sensitivity and specificity of current diagnostic methods. To maintain regular testing quality, it is recommended that a quality control system be implemented in the future, employing blind serum testing.

Moreover, the current scoring system for AIH fibrosis is mainly derived from viral hepatitis research. Therefore, deepening the recent AIH research is expected to establish a unified histological scoring system to quantify AIH fibrosis in the future. AIH patients face significant unmet clinical needs, and treating those who are intolerant or refractory to standard therapy is challenging. Future treatments should differentiate AIH from other autoimmune diseases, focusing precisely on AIH pathways to define more specific therapies. In parallel, more descriptions of the pathogenesis of AIH should be documented to address treatment options further. Aberrant immune regulatory pathways, epigenetic genome, and intestinal microecology are essential therapeutic targets to alter AIH treatment based on an increasing understanding of disease pathogenesis. The treatment of AIH will be able to achieve the effect of removing pathogenic autoimmune cells while retaining autoprotective immunity. In order to attain this objective, it is imperative to thoroughly investigate the existing body of research about the underlying mechanism of AIH. By adopting this approach, we can better understand genetics, facilitating the implementation of genuinely personalized medical interventions. A comprehensive comprehension of triggers, immunopathogenic mechanisms, and effector processes can promote the advancement of improved and more diagnostic experimental approaches and the formulation of novel therapeutic strategies. Eventually, the tolerance of liver autoantigens will be restored, thereby changing our current treatment plan, providing patients with a better quality of life, and prolonging the prognosis of patients.

## References

[1] Kwon HJ, Won YS, Park O, Feng D, Gao B. Opposing effects of prednisolone treatment on T/NKT cell- and hepatotoxin-mediated hepatitis in mice. Hepatology. 2014;59:1094–106. 10.1002/hep.26748 PMC3943761 24115096

[2] de Campos Mazo DF, de Vasconcelos GB, Pereira MA, de Mello ES, Bacchella T, Carrilho FJ, et al. Clinical spectrum and therapeutic approach to hepatocellular injury in patients with hyperthyroidism. Clin Exp Gastroenterol. 2013;6:9–17. 10.2147/CEG.S39358 PMC3579408 23550044

[3] Ferri Liu PM, de Miranda DM, Fagundes ED, Ferreira AR. Simões e Silva AC: Autoimmune hepatitis in childhood: the role of genetic and immune factors. World J Gastroenterol. 2013;19:4455–63. 10.3748/wjg.v19.i28.4455 PMC3725369 23901220

[4] Hu W, Wu XJ, Ni YJ, Hao HR, Yu WN, Zhou HW. Metabolic syndrome is independently associated with a mildly reduced estimated glomerular filtration rate: a cross-sectional study. BMC Nephrol. 2017;18:192. 10.1186/s12882-017-0597-3 PMC5470228 28610620

[5] Nunna V, Jalal N, Bureik M. Anti-CYP4Z1 autoantibodies detected in breast cancer patients. Cell Mol Immunol. 2017;14:572–4. 10.1038/cmi.2017.21 PMC5518819 28435160

[6] Ertekin V, Tosun MS, Selimoglu MA. Is there need for a new hepatitıs B vaccine schedule for children with celiac disease? Hepat Mon. 2011;11:634–7. 10.5812/kowsar.1735143X.715 PMC3227494 22140387

[7] Gelber AC, Manno RL, Shah AA, Woods A, Le EN, Boin F, et al. Race and association with disease manifestations and mortality in scleroderma: a 20-year experience at the Johns Hopkins Scleroderma Center and review of the literature. Medicine (Baltimore). 2013;92:191–205. 10.1097/MD.0b013e31829be125 PMC4553970 23793108

[8] Liu X, Qin S. Immune Checkpoint Inhibitors in Hepatocellular Carcinoma: Opportunities and Challenges. Oncologist. 2019;24:S3–10. 10.1634/theoncologist.2019-IO-S1-s01 PMC6394775 30819826

[9] Kiyokawa H, Yasuda H. Serum monomeric laminin-γ2 as a novel biomarker for hepatocellular carcinoma. Cancer Sci. 2017;108:1432–9. 10.1111/cas.13261 PMC5497925 28418226

[10] Wang G, Wang H, Singh S, Zhou P, Yang S, Wang Y, et al. ADAR1 prevents liver injury from inflammation and suppresses interferon production in hepatocytes. Am J Pathol. 2015;185:3224–37. 10.1016/j.ajpath.2015.08.002 PMC4729276 26453800

[11] Mirabella M, Taramasso L, Nicolini LA, Russo R, Viscoli C, Di Biagio A. Successful recovery of associated interstitial nephritis and focal segmental glomerulosclerosis in patients with HCV and HIV treated with sofosbuvir and daclatasvir and revision of literature. Clin Nephrol Case Stud. 2018;6:31–5. 10.5414/CNCS109221 PMC6218875 30406000

[12] Kaur N, Minz RW, Anand S, Saikia B, Aggarwal R, Das A, et al. HLA DRB1 alleles discriminate the manifestation of autoimmune hepatitis as type 1 or type 2 in North Indian population. J Clin Exp Hepatol. 2014;4:14–8. 10.1016/j.jceh.2013.12.002 PMC4017209 25755530

[13] Li Y, Wang W, Tang L, He X, Yan X, Zhang X, et al. Chemokine (C-X-C motif) ligand 13 promotes intrahepatic chemokine (C-X-C motif) receptor 5 + lymphocyte homing and aberrant B-cell immune responses in primary biliary cirrhosis. Hepatology. 2015;61:1998–2007. 10.1002/hep.27725 PMC4441570 25627620

[14] Ollila HM, Ravel JM, Han F, Faraco J, Lin L, Zheng X, et al. HLA-DPB1 and HLA class I confer risk of and protection from narcolepsy. Am J Hum Genet. 2015;96:136–46. 10.1016/j.ajhg.2014.12.010 PMC4289679 25574827

[15] Mieli-Vergani G, Vergani D, Czaja AJ, Manns MP, Krawitt EL, Vierling JM, et al. Autoimmune hepatitis. Nat Rev Dis Primers. 2018;4:18017. 10.1038/nrdp.2018.17 29644994

[16] Doherty DG, Donaldson PT, Underhill JA, Farrant JM, Duthie A, Mieli-Vergani G, et al. Allelic sequence variation in the HLA class II genes and proteins in patients with autoimmune hepatitis. Hepatology. 1994;19:609–15. 10.1002/hep.1840190311 8119685

[17] van Gerven NM, de Boer YS, Zwiers A, Verwer BJ, Drenth JP, van Hoek B, et al. HLA-DRB1*03:01 and HLA-DRB1*04:01 modify the presentation and outcome in autoimmune hepatitis type-1. Genes Immun. 2015;16:247–52. 10.1038/gene.2014.82 25611558

[18] Donaldson PT. Genetics of liver disease: immunogenetics and disease pathogenesis. Gut. 2004;53:599–608. 10.1136/gut.2003.031732 PMC1773998 15016758

[19] Elfaramawy AA, Elhossiny RM, Abbas AA, Aziz HM. HLA-DRB1 as a risk factor in children with autoimmune hepatitis and its relation to hepatitis A infection. Ital J Pediatr. 2010;36:73. 10.1186/1824-7288-36-73 PMC2992538 21067577

[20] Ma Y, Bogdanos DP, Hussain MJ, Underhill J, Bansal S, Longhi MS, et al. Polyclonal T-cell responses to cytochrome P450IID6 are associated with disease activity in autoimmune hepatitis type 2. Gastroenterology. 2006;130:868–82. 10.1053/j.gastro.2005.12.020 16530525

[21] Huang AC, Postow MA, Orlowski RJ, Mick R, Bengsch B, Manne S, et al. T-cell invigoration to tumour burden ratio associated with anti-PD-1 response. Nature. 2017;545:60–5. 10.1038/nature22079 PMC5554367 28397821

[22] Agarwal K, Czaja AJ, Jones DE, Donaldson PT. Cytotoxic T lymphocyte antigen-4 (CTLA-4) gene polymorphisms and susceptibility to type 1 autoimmune hepatitis. Hepatology. 2000;31:49–53. 10.1002/hep.510310110 10613727

[23] Ahuja N, Singh J, Minz RW, Anand S, Das A, Taneja S. HLA and non-HLA gene polymorphisms in autoimmune hepatitis patients of North Indian adults. Front Immunol. 2022;13:984083. 10.3389/fimmu.2022.984083 PMC9891307 36741403

[24] Chaouali M, Carvalho A, Tezeghdenti A, Ben Azaiez M, Cunha C, Ghazouani E, et al. Cytotoxic T lymphocyte antigen-4 gene polymorphisms and susceptibility to type 1 autoimmune hepatitis in the Tunisian population. Genes Dis. 2018;5:256–62. 10.1016/j.gendis.2017.12.006 PMC6176120 30320190

[25] Xu E, Cao H, Lin L, Liu H. rs10499194 polymorphism in the tumor necrosis factor-α inducible protein 3 (TNFAIP3) gene is associated with type-1 autoimmune hepatitis risk in Chinese Han population. PLoS One. 2017;12:e0176471. 10.1371/journal.pone.0176471 PMC5407796 28448618

[26] Hersh AO, Prahalad S. Immunogenetics of juvenile idiopathic arthritis: a comprehensive review. J Autoimmun. 2015;64:113–24. 10.1016/j.jaut.2015.08.002 PMC4838197 26305060

[27] Higuchi T, Oka S, Furukawa H. Role of deleterious single nucleotide variants in the coding regions of TNFAIP3 for Japanese autoimmune hepatitis with cirrhosis. Sci Rep. 2019;9:7925. 10.1038/s41598-019-44524-5 PMC6538649 31138864

[28] Zwolak A, Surdacka A, Daniluk J. Bcl-2 and Fas expression in peripheral blood leukocytes of patients with alcoholic and autoimmune liver disorders. Hum Exp Toxicol. 2016;35:799–807. 10.1177/0960327115607078 26429926

[29] Ogawa S, Sakaguchi K, Takaki A, Shiraga K, Sawayama T, Mouri H, et al. Increase in CD95 (Fas/APO-1)-positive CD4 + and CD8 + T cells in peripheral blood derived from patients with autoimmune hepatitis or chronic hepatitis C with autoimmune phenomena. J Gastroenterol Hepatol. 2000;15:69–75. 10.1046/j.1440-1746.2000.02044.x 10719750

[30] Guicciardi ME, Gores GJ. Apoptosis: a mechanism of acute and chronic liver injury. Gut. 2005;54:1024–33. 10.1136/gut.2004.053850 PMC1774601 15951554

[31] Tam J, Liu J, Mukhopadhyay B, Cinar R, Godlewski G, Kunos G. Endocannabinoids in liver disease. Hepatology. 2011;53:346–55. 10.1002/hep.24077 PMC3073545 21254182

[32] Tampa M, Mitran MI, Mitran CI. Mediators of inflammation - a potential source of biomarkers in oral squamous cell carcinoma. J Immunol Res. 2018;2018:1061780. 10.1155/2018/1061780 PMC6260538 30539028

[33] Abe K, Takahashi A, Fujita M, Hayashi M, Okai K, Nozawa Y, et al. Interleukin-33/ST2-mediated inflammation plays a critical role in the pathogenesis and severity of type I autoimmune hepatitis. Hepatol Commun. 2019;3:670–84. 10.1002/hep4.1326 PMC6492473 31061955

[34] Luan J, Zhang X, Wang S, Li Y, Fan J, Chen W, et al. NOD-like receptor protein 3 inflammasome-dependent IL-1β accelerated ConA-induced hepatitis. Front Immunol. 2018;9:758. 10.3389/fimmu.2018.00758 PMC5902503 29692782

[35] Jiang R, Tang J, Zhang X, He Y, Yu Z, Chen S, et al. CCN1 promotes inflammation by inducing IL-6 production via α6β1/PI3K/Akt/NF-κB pathway in autoimmune hepatitis. Front Immunol. 2022;13:810671. 10.3389/fimmu.2022.810671 PMC9084230 35547732

[36] Tu H, Chen D, Cai C, Du Q, Lin H, Pan T, et al. microRNA-143-3p attenuated development of hepatic fibrosis in autoimmune hepatitis through regulation of TAK1 phosphorylation. J Cell Mol Med. 2020;24:1256–67. 10.1111/jcmm.14750 PMC6991639 31808606

[37] Cai C, Chen DZ, Tu HX, Chen WK, Ge LC, Fu TT, et al. MicroRNA-29c acting on FOS plays a significant role in nonalcoholic Steatohepatitis through the Interleukin-17 signaling pathway. Front Physiol. 2021;12:597449. 10.3389/fphys.2021.597449 PMC8078210 33927635

[38] Tu Y, Chen D, Pan T, Chen Z, Xu J, Jin L, et al. Inhibition of miR-431-5p attenuated liver apoptosis through KLF15/p53 signal pathway in S100 induced autoimmune hepatitis mice. Life Sci. 2021;280:119698. 10.1016/j.lfs.2021.119698 34111466

[39] Yamaura Y, Tatsumi N, Takagi S, Tokumitsu S, Fukami T, Tajiri K, et al. Serum microRNA profiles in patients with chronic hepatitis B, chronic hepatitis C, primary biliary cirrhosis, autoimmune hepatitis, nonalcoholic steatohepatitis, or drug-induced liver injury. Clin Biochem. 2017;50:1034–9. 10.1016/j.clinbiochem.2017.08.010 28823616

[40] Abe K, Suzuki R, Fujita M, Hayashi M, Takahashi A, Ohira H. Circulating extracellular vesicle-encapsulated microRNA-557 induces a proinflammatory immune response and serves as a diagnostic or relapse marker in autoimmune hepatitis. Hepatol Res. 2022;52:1034–49. 10.1111/hepr.13829 35962993

[41] Huang C, Xing X, Xiang X, Fan X, Men R, Ye T, et al. MicroRNAs in autoimmune liver diseases: from diagnosis to potential therapeutic targets. Biomed Pharmacother. 2020;130:110558. 10.1016/j.biopha.2020.110558 32781357

[42] Caparrós E, Juanola O, Gómez-Hurtado I, Puig-Kroger A, Piñero P, Zapater P, et al. Liver sinusoidal endothelial cells contribute to hepatic antigen-presenting cell function and Th17 expansion in cirrhosis. Cells. 2020;9:1227. 10.3390/cells9051227 PMC7290576 32429209

[43] Palazon A, Goldrath AW, Nizet V, Johnson RS. HIF transcription factors, inflammation, and immunity. Immunity. 2014;41:518–28. 10.1016/j.immuni.2014.09.008 PMC4346319 25367569

[44] Patten DA, Wilson GK, Bailey D, Shaw RK, Jalkanen S, Salmi M, et al. Human liver sinusoidal endothelial cells promote intracellular crawling of lymphocytes during recruitment: a new step in migration. Hepatology. 2017;65:294–309. 10.1002/hep.28879 PMC5321563 27770554

[45] Burghardt S, Claass B, Erhardt A, Karimi K, Tiegs G. Hepatocytes induce Foxp3⁺ regulatory T cells by Notch signaling. J Leukoc Biol. 2014;96:571–7. 10.1189/jlb.2AB0613-342RR 24970859

[46] Bowen DG, Zen M, Holz L, Davis T, McCaughan GW, Bertolino P. The site of primary T cell activation is a determinant of the balance between intrahepatic tolerance and immunity. J Clin Invest. 2004;114:701–12. 10.1172/JCI21593 PMC514586 15343389

[47] Béland K, Marceau G, Labardy A, Bourbonnais S, Alvarez F. Depletion of B cells induces remission of autoimmune hepatitis in mice through reduced antigen presentation and help to T cells. Hepatology. 2015;62:1511–23. 10.1002/hep.27991 26175263

[48] Ponziani FR, Nicoletti A, Gasbarrini A, Pompili M. Diagnostic and therapeutic potential of the gut microbiota in patients with early hepatocellular carcinoma. Ther Adv Med Oncol. 2019;11:1758835919848184. 10.1177/1758835919848184 PMC6535703 31205505

[49] Tan M, Mosaoa R, Graham GT. Inhibition of the mitochondrial citrate carrier, Slc25a1, reverts steatosis, glucose intolerance, and inflammation in preclinical models of NAFLD/NASH. Cell Death Differ. 2020;27:2143–57. 10.1038/s41418-020-0491-6 PMC7308387 31959914

[50] Yi J, Jung J, Hong SW, Lee JY, Han D, Kim KS, et al. Unregulated antigen-presenting cell activation by T cells breaks self tolerance. Proc Natl Acad Sci. 2019;116:1007–16. 10.1073/pnas.1818624116 PMC6338877 30598454

[51] Mamlouk O, Lin JS. Checkpoint inhibitor-related renal vasculitis and use of rituximab. J Immunother cancer. 2020;8:8. 10.1136/jitc-2020-000750 PMC7380836 32718987

[52] Tagliamonte M, Petrizzo A, Mauriello A, Tornesello ML, Buonaguro FM, Buonaguro L. Potentiating cancer vaccine efficacy in liver cancer. Oncoimmunology. 2018;7:e1488564. 10.1080/2162402X.2018.1488564 PMC6169594 30288355

[53] Narayan K, Sylvia KE, Malhotra N, Yin CC, Martens G, Vallerskog T, et al. Intrathymic programming of effector fates in three molecularly distinct γδ T cell subtypes. Nat Immunol. 2012;13:511–8. 10.1038/ni.2247 PMC3427768 22473038

[54] Cheng M, Hu S. Lung-resident γδ T cells and their roles in lung diseases. Immunology. 2017;151:375–84. 10.1111/imm.12764 PMC5506441 28555812

[55] Crawshaw A, Kendrick YR, McMichael AJ, Ho LP. Abnormalities in iNKT cells are associated with impaired ability of monocytes to produce IL-10 and suppress T-cell proliferation in sarcoidosis. Eur J Immunol. 2014;44:2165–74. 10.1002/eji.201344284 PMC4746743 24723379

[56] Nishio K, Miyagi T, Tatsumi T, Mukai K, Yokoyama Y, Yoshioka T, et al. Invariant natural killer T cell deficiency leads to the development of spontaneous liver inflammation dependent on γδT cells in mice. J Gastroenterol. 2015;50:1124–33. 10.1007/s00535-015-1060-5 25791517

[57] Hayday A, Tigelaar R. Immunoregulation in the tissues by gammadelta T cells. Nat Rev Immunol. 2003;3:233–42. 10.1038/nri1030 12658271

[58] Martins EB, Graham AK, Chapman RW, Fleming KA. Elevation of gamma delta T lymphocytes in peripheral blood and livers of patients with primary sclerosing cholangitis and other autoimmune liver diseases. Hepatology. 1996;23:988–93. 10.1002/hep.510230508 8621180

[59] Chauhan SK, Tripathy NK, Sinha N, Nityanand S. T-cell receptor repertoire of circulating gamma delta T-cells in Takayasu’s arteritis. Clin Immunol. 2006;118:243–9. 10.1016/j.clim.2005.10.010 16307908

[60] Giacomelli R, Matucci-Cerinic M, Cipriani P, Ghersetich I, Lattanzio R, Pavan A, et al. Circulating Vdelta1 + T cells are activated and accumulate in the skin of systemic sclerosis patients. Arthritis Rheum. 1998;41:327–34. 10.1002/1529-0131(199802)41:2<327::AID-ART17>3.0.CO;2-S 9485091

[61] Ferri S, Longhi MS, De Molo C, Lalanne C, Muratori P, Granito A, et al. A multifaceted imbalance of T cells with regulatory function characterizes type 1 autoimmune hepatitis. Hepatology. 2010;52:999–1007. 10.1002/hep.23792 20683931

[62] He Q, Lu Y, Tian W, Jiang R, Yu W, Liu Y, et al. TOX deficiency facilitates the differentiation of IL-17A-producing γδ T cells to drive autoimmune hepatitis. Cell Mol Immunol. 2022;19:1102–16. 10.1038/s41423-022-00912-y PMC9508111 35986136

[63] Aoki T, Motohashi S, Koseki H. Regeneration of invariant natural killer T (iNKT) cells: application of iPSC technology for iNKT cell-targeted tumor immunotherapy. Inflamm Regen. 2023;43:27. 10.1186/s41232-023-00275-5 PMC10176773 37170375

[64] Grant CR, Liberal R, Holder BS, Cardone J, Ma Y, Robson SC, et al. Dysfunctional CD39(POS) regulatory T cells and aberrant control of T-helper type 17 cells in autoimmune hepatitis. Hepatology. 2014;59:1007–15. 10.1002/hep.26583 PMC6377365 23787765

[65] Liberal R, Longhi MS, Mieli-Vergani G, Vergani D. Pathogenesis of autoimmune hepatitis. Best Pract Res Clin Gastroenterol. 2011;25:653–64. 10.1016/j.bpg.2011.09.009 22117632

[66] Liaskou E, Hirschfield GM, Gershwin ME. Mechanisms of tissue injury in autoimmune liver diseases. Semin Immunopathol. 2014;36:553–68. 10.1007/s00281-014-0439-3 25082647

[67] Zhao L, Tang Y, You Z, Wang Q, Liang S, Han X, et al. Interleukin-17 contributes to the pathogenesis of autoimmune hepatitis through inducing hepatic interleukin-6 expression. PLoS One. 2011;6:e18909. 10.1371/journal.pone.0018909 PMC3079758 21526159

[68] Harrington C, Krishnan S, Mack CL, Cravedi P, Assis DN, Levitsky J. Noninvasive biomarkers for the diagnosis and management of autoimmune hepatitis. Hepatology. 2022;76:1862–79. 10.1002/hep.32591 PMC9796683 35611859

[69] Yuksel M, Wang Y, Tai N, Peng J, Guo J, Beland K, et al. A novel “humanized mouse” model for autoimmune hepatitis and the association of gut microbiota with liver inflammation. Hepatology. 2015;62:1536–50. 10.1002/hep.27998 PMC4763614 26185095

[70] Katz Sand I, Zhu Y, Ntranos A, Clemente JC, Cekanaviciute E, Brandstadter R, et al. Disease-modifying therapies alter gut microbial composition in MS. Neurol Neuroimmunol Neuroinflamm. 2019;6:e517. 10.1212/NXI.0000000000000517 PMC6278850 30568995

[71] Bezerra JA, Spino C, Magee JC, Shneider BL, Rosenthal P, Wang KS, et al. Use of corticosteroids after hepatoportoenterostomy for bile drainage in infants with biliary atresia: the START randomized clinical trial. Jama. 2014;311:1750–9. 10.1001/jama.2014.2623 PMC4303045 24794368

[72] Maggiore G, Veber F, Bernard O, Hadchouel M, Homberg JC, Alvarez F, et al. Autoimmune hepatitis associated with anti-actin antibodies in children and adolescents. J Pediatr Gastroenterol Nutr. 1993;17:376–81. 10.1097/00005176-199311000-00007 8145091

[73] Gregorio GV, Portmann B, Reid F, Donaldson PT, Doherty DG, McCartney M, et al. Autoimmune hepatitis in childhood: a 20-year experience. Hepatology. 1997;25:541–7. 10.1002/hep.510250308 9049195

[74] Keating JJ, O'brien CJ, Stellon AJ, Portmann BC, Johnson RD, Johnson PJ, et al. Influence of aetiology, clinical and histological features on survival in chronic active hepatitis: an analysis of 204 patients. Q J Med. 1987;62:59–66. 3423206

[75] Zhong B, Wang Y, Zhang G, Wang Z. Environmental iodine content, female sex and age are associated with new-onset amiodarone-induced hypothyroidism: a systematic review and meta-analysis of adverse reactions of amiodarone on the thyroid. Cardiology. 2016;134:366–71. 10.1159/000444578 27100205

[76] Singh S, Venkatesh SK, Wang Z, Miller FH, Motosugi U, Low RN, et al. Diagnostic performance of magnetic resonance elastography in staging liver fibrosis: a systematic review and meta-analysis of individual participant data. Clin Gastroenterol Hepatol. 2015;13(440–451):e446. 10.1016/j.cgh.2014.09.046 PMC4333001 25305349

[77] Paiardini A, Pascarella S. Structural mimicry between SLA/LP and Rickettsia surface antigens as a driver of autoimmune hepatitis: insights from an in silico study. Theor Biol Med Model. 2013;10:25. 10.1186/1742-4682-10-25 PMC3636016 23575112

[78] Ngu JH, Gearry RB, Frampton CM, Stedman CA. Autoimmune hepatitis: the role of environmental risk factors: a population-based study. Hepatol Int. 2013;7:869–75. 10.1007/s12072-013-9448-x 26201924

[79] Rigopoulou EI, Gatselis N, Arvaniti P, Koukoulis GK, Dalekos GN. Alcoholic liver disease and autoimmune hepatitis: sometimes a closer look under the surface is needed. Eur J Intern Med. 2021;85:86–91. 10.1016/j.ejim.2020.12.024 33451888

[80] Dadabhai AS, Saberi B, Lobner K, Shinohara RT, Mullin GE. Influence of vitamin D on liver fibrosis in chronic hepatitis C: A systematic review and meta-analysis of the pooled clinical trials data. World J Hepatol. 2017;9:278–87. 10.4254/wjh.v9.i5.278 PMC5316848 28261385

[81] Karki R, Malireddi RKS, Zhu Q. NLRC3 regulates cellular proliferation and apoptosis to attenuate the development of colorectal cancer. Cell Cycle (Georgetown, Tex). 2017;16:1243–51. 10.1080/15384101.2017.1317414 PMC5531621 28598238

[82] Wei Y, Li Y, Yan L, Sun C, Miao Q, Wang Q, et al. Alterations of gut microbiome in autoimmune hepatitis. Gut. 2020;69:569–77. 10.1136/gutjnl-2018-317836 31201284

[83] Liu Q, Tian H, Kang Y, Tian Y, Li L, Kang X, et al. Probiotics alleviate autoimmune hepatitis in mice through modulation of gut microbiota and intestinal permeability. J Nutr Biochem. 2021;98:108863. 10.1016/j.jnutbio.2021.108863 34517094

[84] Liwinski T, Casar C, Ruehlemann MC, Bang C, Sebode M. A disease-specific decline of the relative abundance of Bifidobacterium in patients with autoimmune hepatitis. Aliment Pharmacol Ther. 2020;51:1417–28. 10.1111/apt.15754 32383181

[85] Liang M, Liwen Z, Jianguo S, Juan D, Fei D, Yin Z, et al. Fecal microbiota transplantation controls progression of experimental autoimmune hepatitis in mice by modulating the TFR/TFH immune imbalance and intestinal microbiota composition. Front Immunol. 2021;12:728723. 10.3389/fimmu.2021.728723 PMC8667314 34912328

[86] Zhang X, Gu S, You L, Xu Y, Zhou D, Chen Y, et al. Gut microbiome and metabolome were altered and strongly associated with platelet count in adult patients with primary immune thrombocytopenia. Front Microbiol. 2020;11:1550. 10.3389/fmicb.2020.01550 PMC7360729 32733424

[87] Lin R, Zhou L, Zhang J, Wang B. Abnormal intestinal permeability and microbiota in patients with autoimmune hepatitis. Int J Clin Exp Pathol. 2015;8:5153–60. PMC4503083 26191211

[88] Arase Y, Shiraishi K, Anzai K, Sato H, Teramura E, Tsuruya K, et al. Effect of sodium glucose co-transporter 2 inhibitors on liver fat mass and body composition in patients with nonalcoholic fatty liver disease and type 2 diabetes Mellitus. Clin Drug Investig. 2019;39:631–41. 10.1007/s40261-019-00785-6 PMC6593121 30993553

[89] Russo MW, Hoofnagle JH, Gu J, Fontana RJ, Barnhart H, Kleiner DE, et al. Spectrum of statin hepatotoxicity: experience of the drug-induced liver injury network. Hepatology. 2014;60:679–86. 10.1002/hep.27157 PMC4110177 24700436

[90] Lee C, Jeong H, Lee KH, Park S, Gang MJ, Bae SK, et al. Evaluation of the efficacy and safety of the herbal formula PM014 in a Cisplatin- and Paclitaxel-treated tumor-bearing mouse model. Integr Cancer Ther. 2020;19:1534735420924711. 10.1177/1534735420924711 PMC7323267 32590912

[91] Tan CK, Ho D, Wang LM, Kumar R. Drug-induced autoimmune hepatitis: a minireview. World J Gastroenterol. 2022;28:2654–66. 10.3748/wjg.v28.i24.2654 PMC9260871 35979160

[92] Guabiraba R, Ryffel B. Dengue virus infection: current concepts in immune mechanisms and lessons from murine models. Immunology. 2014;141:143–56. 10.1111/imm.12188 PMC3904235 24182427

[93] Huang TH, Chen CC, Liu HM, Lee TY, Shieh SH. Resveratrol pretreatment attenuates concanavalin A-induced hepatitis through reverse of aberration in the immune response and regenerative capacity in aged mice. Sci Rep. 2017;7:2705. 10.1038/s41598-017-02881-z PMC5457448 28578410

[94] Mehta-Shah N, Bartlett NL. Management of relapsed/refractory classical Hodgkin lymphoma in transplant-ineligible patients. Blood. 2018;131:1698–703. 10.1182/blood-2017-09-772681 PMC6536701 29500171

[95] Alzaid F, Lagadec F, Albuquerque M, Ballaire R, Orliaguet L, Hainault I, et al. IRF5 governs liver macrophage activation that promotes hepatic fibrosis in mice and humans. JCI Insight. 2016;1:e88689. 10.1172/jci.insight.88689 PMC5135279 27942586

[96] Guerriero E, Capone F, Accardo M, Sorice A, Costantini M, Colonna G, et al. GPX4 and GPX7 over-expression in human hepatocellular carcinoma tissues. Eur J Histochem. 2015;59:2540. 10.4081/ejh.2015.2540 PMC4698610 26708178

[97] Afsar T, Razak S, Almajwal A. Effect of Acacia hydaspica R. Parker extract on lipid peroxidation, antioxidant status, liver function test and histopathology in doxorubicin treated rats. Lipids Health Dis. 2019;18:126. 10.1186/s12944-019-1051-2 PMC6542101 31142345

[98] Shu CW, Huang CM. HSP70s: From tumor transformation to cancer therapy. Clin Med Oncol. 2008;2:335–45. 10.4137/cmo.s475 PMC3161691 21892295

[99] Arvaniti P, Giannoulis G, Gabeta S, Zachou K, Koukoulis GK, Dalekos GN. Belimumab is a promising third-line treatment option for refractory autoimmune hepatitis. JHEP Rep. 2020;2:100123. 10.1016/j.jhepr.2020.100123 PMC7340979 32671332

[100] Ge Z, Feng Y, Muthupalani S, Whary MT, Versalovic J, Fox JG. Helicobacter hepaticus cholesterol-α-glucosyltransferase is essential for establishing colonization in male A/JCr mice. Helicobacter. 2014;19:280–8. 10.1111/hel.12135 PMC4111802 24853076

[101] Vierling JM. Immunopathogenesis: Insights for current and future therapies. Clin Liver Dis (Hoboken). 2014;3:24–8. 10.1002/cld.309 PMC6567855 31236265

[102] Mukherjee T, Khan ID, Guha R, Ganguly T. Cholemic nephrosis (bile cast nephropathy) with severe liver dysfunction. Med J Armed Forces India. 2019;75:216–8. 10.1016/j.mjafi.2018.03.012 PMC6495314 31065193

[103] Adriaanse MPM, Mubarak A, Riedl RG, Ten Kate FJW, Damoiseaux J, Buurman WA, et al. Progress towards non-invasive diagnosis and follow-up of celiac disease in children; a prospective multicentre study to the usefulness of plasma I-FABP. Sci Rep. 2017;7:8671. 10.1038/s41598-017-07242-4 PMC5561259 28819290

[104] Ye J, Hu X, Wu T, Wu Y, Shao C, Li F, et al. Insulin resistance exhibits varied metabolic abnormalities in nonalcoholic fatty liver disease, chronic hepatitis B and the combination of the two: a cross-sectional study. Diabetol Metab Syndr. 2019;11:45. 10.1186/s13098-019-0440-z PMC6570947 31223344

[105] Gruszewska E, Cylwik B, Panasiuk A, Szmitkowski M, Flisiak R, Chrostek L. Total and free serum sialic acid concentration in liver diseases. Biomed Res Int. 2014;2014:876096. 10.1155/2014/876096 PMC4052165 24959592

[106] Sherigar JM, Yavgeniy A, Guss D, Ngo N, Mohanty S. Seronegative autoimmune hepatitis A clinically challenging difficult diagnosis. Case Rep Med. 2017;2017:3516234. 10.1155/2017/3516234 PMC5518508 28761444

[107] Geller SA. Autoimmune hepatitis: histopathology. Clin Liver Dis (Hoboken). 2014;3:19–23. 10.1002/cld.301 PMC6567856 31236264

[108] Béland K, Lapierre P, Alvarez F. Influence of genes, sex, age and environment on the onset of autoimmune hepatitis. World J Gastroenterol. 2009;15:1025–34. 10.3748/wjg.15.1025 PMC2655185 19266593

[109] Deutsch M, Emmanuel T, Koskinas J. Autoimmune Hepatitis or Wilson’s disease, a clinical dilemma. Hepat Mon. 2013;13:e7872. 10.5812/hepatmon.7872 PMC3732661 23922560

[110] Scott J, Gollan JL, Samourian S, Sherlock S. Wilson’s disease, presenting as chronic active hepatitis. Gastroenterology. 1978;74:645–51. 75819

[111] Sebode M, Hartl J, Vergani D, Lohse AW. Autoimmune hepatitis: from current knowledge and clinical practice to future research agenda. Liver Int. 2018;38:15–22. 10.1111/liv.13458 28432836

[112] Woimant F, Djebrani-Oussedik N, Poujois A. New tools for Wilson’s disease diagnosis: exchangeable copper fraction. Ann Transl Med. 2019;7:S70. 10.21037/atm.2019.03.02 PMC6531656 31179307

[113] Fontana RJ, Seeff LB, Andrade RJ, Björnsson E, Day CP, Serrano J, et al. Standardization of nomenclature and causality assessment in drug-induced liver injury: summary of a clinical research workshop. Hepatology. 2010;52:730–42. 10.1002/hep.23696 PMC3616501 20564754

[114] Febres-Aldana CA, Alghamdi S, Krishnamurthy K, Poppiti RJ. Liver fibrosis helps to distinguish autoimmune hepatitis from DILI with autoimmune features: a review of twenty cases. J Clin Transl Hepatol. 2019;7:21–6. 10.14218/JCTH.2018.00053 PMC6441645 30944815

[115] Yatsuji S, Hashimoto E, Kaneda H, Taniai M, Tokushige K, Shiratori K. Diagnosing autoimmune hepatitis in nonalcoholic fatty liver disease: is the International Autoimmune Hepatitis Group scoring system useful? J Gastroenterol. 2005;40:1130–8. 10.1007/s00535-005-1711-z 16378177

[116] Torres Vidal A, Medintz IL. DNA microsystems for biodiagnosis. Micromachines. 2020;11:11. 10.3390/mi11040445 PMC7231314 32340280

[117] Shafiei M, Alavian SM. Autoimmune hepatitis in Iran: what we know, what we don’t know and requirements for better management. Hepat Mon. 2012;12:73–6. 10.5812/hepatmon.840 PMC3321324 22509182

[118] Zhu B, You SL, Wan ZH, Liu HL, Rong YH, Zang H, et al. Clinical characteristics and corticosteroid therapy in patients with autoimmune-hepatitis-induced liver failure. World J Gastroenterol. 2014;20:7473–9. 10.3748/wjg.v20.i23.7473 PMC4064093 24966618

[119] Radzimski C, Probst C, Teegen B, Rentzsch K, Blöcker IM, Dähnrich C, et al. Development of a recombinant cell-based indirect immunofluorescence assay for the determination of autoantibodies against soluble liver antigen in autoimmune hepatitis. Clin Dev Immunol. 2013;2013:572815. 10.1155/2013/572815 PMC3562650 23401700

[120] Arends JE, Ghisetti V, Irving W, Dalton HR, Izopet J, Hoepelman AI, et al. Hepatitis E: an emerging infection in high income countries. J Clin Virol. 2014;59:81–8. 10.1016/j.jcv.2013.11.013 24388207

[121] Vergani D, Alvarez F, Bianchi FB, Cançado EL, Mackay IR, Manns MP, et al. Liver autoimmune serology: a consensus statement from the committee for autoimmune serology of the international autoimmune hepatitis group. J Hepatol. 2004;41:677–83. 10.1016/j.jhep.2004.08.002 15464251

[122] Baeres M, Herkel J, Czaja AJ, Wies I, Kanzler S, Cancado EL, et al. Establishment of standardised SLA/LP immunoassays: specificity for autoimmune hepatitis, worldwide occurrence, and clinical characteristics. Gut. 2002;51:259–64. 10.1136/gut.51.2.259 PMC1773315 12117891

[123] Yeoman AD, Westbrook RH, Zen Y, Bernal W, Al-Chalabi T, Wendon JA, et al. Prognosis of acute severe autoimmune hepatitis (AS-AIH): the role of corticosteroids in modifying outcome. J Hepatol. 2014;61:876–82. 10.1016/j.jhep.2014.05.021 24842305

[124] Motawi TK, El-Maraghy SA, Sharaf SA, Said SE. Association of CARD10 rs6000782 and TNF rs1799724 variants with paediatric-onset autoimmune hepatitis. J Adv Res. 2019;15:103–10. 10.1016/j.jare.2018.10.001 PMC6300463 30581618

[125] Saremi L, Lotfipanah S, Mohammadi M, Hosseinzadeh H, Fathi-Kazerooni M, Johari B, et al. The Pro12Ala polymorphism in the PPAR-γ2 gene is not associated with an increased risk of NAFLD in Iranian patients with type 2 diabetes mellitus. Cell Mol Biol Lett. 2019;24:12. 10.1186/s11658-019-0138-0 PMC6419465 30923554

[126] EASL clinical practice guidelines: autoimmune hepatitis. J Hepatol. 2015;63:971–1004. 10.1016/j.jhep.2015.06.030 26341719

[127] Doycheva I, Watt KD, Gulamhusein AF. Autoimmune hepatitis: current and future therapeutic options. Liver Int. 2019;39:1002–13. 10.1111/liv.14062 30716203

[128] Gu L, Deng WS, Sun XF, Zhou H, Xu Q. Rapamycin ameliorates CCl4-induced liver fibrosis in mice through reciprocal regulation of the Th17/Treg cell balance. Mol Med Rep. 2016;14:1153–61. 10.3892/mmr.2016.5392 PMC4940054 27315465

[129] Geetha D, Specks U, Stone JH, Merkel PA, Seo P, Spiera R, et al. Rituximab versus cyclophosphamide for ANCA-associated vasculitis with renal involvement. J Am Soc Nephrol. 2015;26:976–85. 10.1681/ASN.2014010046 PMC4378104 25381429

[130] Qiu S, Deng L, Liao X, Nie L, Qi F, Jin K, et al. Tumor-associated macrophages promote bladder tumor growth through PI3K/AKT signal induced by collagen. Cancer Sci. 2019;110:2110–8. 10.1111/cas.14078 PMC6609800 31120174

[131] Dyson JK, Wong LL, Bigirumurame T, Hirschfield GM, Kendrick S, Oo YH, et al. Inequity of care provision and outcome disparity in autoimmune hepatitis in the United Kingdom. Aliment Pharmacol Ther. 2018;48:951–60. 10.1111/apt.14968 PMC6667893 30226274

[132] Thurairajah PH, Carbone M, Bridgestock H, Thomas P, Hebbar S, Gunson BK, et al. Late acute liver allograft rejection; a study of its natural history and graft survival in the current era. Transplantation. 2013;95:955–9. 10.1097/TP.0b013e3182845f6c 23442806

[133] Nichols NL, Satriotomo I, Allen LL. Mechanisms of Enhanced Phrenic Long-Term Facilitation in SOD1(G93A) Rats. J Neurosci: Off J Soc Neurosci. 2017;37:5834–45. 10.1523/JNEUROSCI.3680-16.2017 PMC5473203 28500219

[134] Schramm C, Wahl I, Weiler-Normann C, Voigt K, Wiegard C, Glaubke C, et al. Health-related quality of life, depression, and anxiety in patients with autoimmune hepatitis. J Hepatol. 2014;60:618–24. 10.1016/j.jhep.2013.10.035 24240053

[135] Johnson PJ, McFarlane IG, Williams R. Azathioprine for long-term maintenance of remission in autoimmune hepatitis. N Engl J Med. 1995;333:958–63. 10.1056/NEJM199510123331502 7666914

[136] Dhaliwal HK, Anderson R, Thornhill EL, Schneider S, McFarlane E, Gleeson D, et al. Clinical significance of azathioprine metabolites for the maintenance of remission in autoimmune hepatitis. Hepatology. 2012;56:1401–8. 10.1002/hep.25760 22488741

[137] Czaja AJ, Davis GL, Ludwig J, Taswell HF. Complete resolution of inflammatory activity following corticosteroid treatment of HBsAg-negative chronic active hepatitis. Hepatology. 1984;4:622–7. 10.1002/hep.1840040409 6745850

[138] Forno E, Lescher R, Strunk R, Weiss S, Fuhlbrigge A, Celedón JC. Decreased response to inhaled steroids in overweight and obese asthmatic children. J Allergy Clin Immunol. 2011;127:741–9. 10.1016/j.jaci.2010.12.010 PMC3056233 21377042

[139] Parker R, Oo YH, Adams DH. Management of patients with difficult autoimmune hepatitis. Ther Adv Gastroenterol. 2012;5:421–37. 10.1177/1756283X12450251 PMC3491680 23152735

[140] Yuan F, Quan LD, Cui L, Goldring SR, Wang D. Development of macromolecular prodrug for rheumatoid arthritis. Adv Drug Deliv Rev. 2012;64:1205–19. 10.1016/j.addr.2012.03.006 PMC3572768 22433784

[141] Hsu WT, Lin CH, Jui HY, Tseng YH, Shun CT, Hsu MC, et al. CXCR4 antagonist reduced the incidence of acute rejection and controlled cardiac allograft vasculopathy in a swine heart transplant model receiving a mycophenolate-based immunosuppressive regimen. Transplantation. 2018;102:2002–11. 10.1097/TP.0000000000002404 PMC6257103 30095739

[142] Lu Y, Bratton S, Heydel JM, Radominska-Pandya A. Effect of retinoids on UDP-glucuronosyltransferase 2B7 mRNA expression in Caco-2 cells. Drug Metab Pharmacokinet. 2008;23:364–72. 10.2133/dmpk.23.364 PMC3129696 18974614

[143] Saitis A, Gatselis N, Zachou K, Dalekos GN. Use of TNFα antagonists in refractory AIH: revealing the unforeseen. J Hepatol. 2013;59:197–8. 10.1016/j.jhep.2013.02.029 23528379

[144] Abdollahi M, Ekrami NK, Ghojazadeh M, Boezen HM, Somi M, Alizadeh BZ. Tacrolimus and mycophenolate mofetil as second-line treatment in autoimmune hepatitis: Is the evidence of sufficient quality to develop recommendations? World J Gastroenterol. 2020;26:5896–910. 10.3748/wjg.v26.i38.5896 PMC7579758 33132643

[145] Madonna R, Van Laake LW, Botker HE, Davidson SM, De Caterina R, Engel FB, et al. ESC working group on cellular biology of the heart: position paper for cardiovascular research: tissue engineering strategies combined with cell therapies for cardiac repair in ischaemic heart disease and heart failure. Cardiovasc Res. 2019;115:488–500. 10.1093/cvr/cvz010 PMC6383054 30657875

[146] Udompap P, Kim D, Kim WR. Current and future burden of chronic nonmalignant liver disease. Clin Gastroenterol Hepatol. 2015;13:2031–41. 10.1016/j.cgh.2015.08.015 PMC4618163 26291665

[147] Zama D, Cocchi I, Masetti R, Specchia F, Alvisi P, Gambineri E, et al. Late-onset of immunodysregulation, polyendocrinopathy, enteropathy, x-linked syndrome (IPEX) with intractable diarrhea. Ital J Pediatr. 2014;40:68. 10.1186/s13052-014-0068-4 PMC4421998 25326164

[148] Geetha D, Levine SM, Manno RL, Valsamakis A, Ghazarian S, Seo P. BK virus replication in patients with anti-neutrophil cytoplasmic antibody-associated vasculitis. Am J Nephrol. 2014;39:20–6. 10.1159/000357409 PMC4142761 24401699

[149] Chi H. Regulation and function of mTOR signalling in T cell fate decisions. Nat Rev Immunol. 2012;12:325–38. 10.1038/nri3198 PMC3417069 22517423

[150] Delgoffe GM, Kole TP, Zheng Y, Zarek PE, Matthews KL, Xiao B, et al. The mTOR kinase differentially regulates effector and regulatory T cell lineage commitment. Immunity. 2009;30:832–44. 10.1016/j.immuni.2009.04.014 PMC2768135 19538929

[151] Delgoffe GM, Pollizzi KN, Waickman AT, Heikamp E, Meyers DJ, Horton MR, et al. The kinase mTOR regulates the differentiation of helper T cells through the selective activation of signaling by mTORC1 and mTORC2. Nat Immunol. 2011;12:295–303. 10.1038/ni.2005 PMC3077821 21358638

[152] Apostolidis SA, Rodríguez-Rodríguez N, Suárez-Fueyo A, Dioufa N, Ozcan E, Crispín JC, et al. Phosphatase PP2A is requisite for the function of regulatory T cells. Nat Immunol. 2016;17:556–64. 10.1038/ni.3390 PMC4837024 26974206

[153] Yang K, Shrestha S, Zeng H, Karmaus PW, Neale G, Vogel P, et al. T cell exit from quiescence and differentiation into Th2 cells depend on Raptor-mTORC1-mediated metabolic reprogramming. Immunity. 2013;39:1043–56. 10.1016/j.immuni.2013.09.015 PMC3986063 24315998

[154] Ouyang X, Han Y, Qu G, Li M, Wu N, Liu H, et al. Metabolic regulation of T cell development by Sin1-mTORC2 is mediated by pyruvate kinase. J Mol Cell Biol. 2019;11:93–106. 10.1093/jmcb/mjy065 PMC6392101 30428057

[155] Lee K, Nam KT, Cho SH, Gudapati P, Hwang Y, Park DS, et al. Vital roles of mTOR complex 2 in Notch-driven thymocyte differentiation and leukemia. J Exp Med. 2012;209:713–28. 10.1084/jem.20111470 PMC3328370 22473959

[156] Haxhinasto S, Mathis D, Benoist C. The AKT-mTOR axis regulates de novo differentiation of CD4+ Foxp3+ cells. J Exp Med. 2008;205:565–74. 10.1084/jem.20071477 PMC2275380 18283119

[157] Chen H, Zhang L, Zhang H, Xiao Y, Shao L, Li H, et al. Disruption of TSC1/2 signaling complex reveals a checkpoint governing thymic CD4+ CD25+ Foxp3+ regulatory T-cell development in mice. Faseb J. 2013;27:3979–90. 10.1096/fj.13-235408 23882125

[158] Kerkar N, Dugan C, Rumbo C, Morotti RA, Gondolesi G, Shneider BL, et al. Rapamycin successfully treats post-transplant autoimmune hepatitis. Am J Transpl. 2005;5:1085–9. 10.1111/j.1600-6143.2005.00801.x 15816890

[159] Czaja AJ. Review article: next-generation transformative advances in the pathogenesis and management of autoimmune hepatitis. Aliment Pharmacol Ther. 2017;46:920–37. 10.1111/apt.14324 28901565

[160] Glassner K, Quigley EM, Franco L, Victor DW. 3rd: Autoimmune liver disease and the enteric microbiome. AIMS Microbiol. 2018;4:334–46. 10.3934/microbiol.2018.2.334 PMC6604930 31294219

[161] Todoric J, Antonucci L, Karin M. Targeting Inflammation in Cancer Prevention and Therapy. Cancer Prev Res (Phila). 2016;9:895–905. 10.1158/1940-6207.CAPR-16-0209 PMC5142754 27913448

[162] Hsu MC, Liu SH, Wang CW, Hu NY, Wu ESC, Shih YC, et al. JKB-122 is effective, alone or in combination with prednisolone in ConA-induced hepatitis. Eur J Pharmacol. 2017;812:113–20. 10.1016/j.ejphar.2017.07.012 28694068

[163] Berzofsky JA, Pendleton CD, Clerici M, Ahlers J, Lucey DR, Putney SD, et al. Construction of peptides encompassing multideterminant clusters of human immunodeficiency virus envelope to induce in vitro T cell responses in mice and humans of multiple MHC types. J Clin Invest. 1991;88:876–84. 10.1172/JCI115389 PMC295474 1715888

[164] Büschenfelde KH, Kössling FK, Miescher PA. Experimental chronic active hepatitis in rabbits following immunization with human liver proteins. Clin Exp Immunol. 1972;11:99–108. PMC1553689 4338952

[165] Qiu F, Tang R, Zuo X, Shi X, Wei Y, Zheng X, et al. A genome-wide association study identifies six novel risk loci for primary biliary cholangitis. Nat Commun. 2017;8:14828. 10.1038/ncomms14828 PMC5429142 28425483

[166] Sundin U, Heigl Z, Sundqvist KG. The IgG antibody reactivity of sera from patients with active chronic hepatitis to a crude liver antigen and liver specific protein (LSP): analysis by ELISA and immunoblotting. Clin Exp Immunol. 1988;74:276–82. PMC1541812 3265656

[167] Tsouris Z, Liaskos C, Dardiotis E, Scheper T, Tsimourtou V, Meyer W, et al. A comprehensive analysis of antigen-specific autoimmune liver disease related autoantibodies in patients with multiple sclerosis. Autoimmun Highlights. 2020;11:7. 10.1186/s13317-020-00130-4 PMC7147023 32308974

[168] Zhu L, Chen D, Zhu Y, Pan T, Xia D, Cai T, et al. GPX4-regulated Ferroptosis mediates S100-induced experimental autoimmune hepatitis associated with the Nrf2/HO-1 signaling pathway. Oxid Med Cell Longev. 2021;2021:6551069. 10.1155/2021/6551069 PMC8712167 34966478

[169] Ferhat MH, Robin A, Barbier L, Thierry A, Gombert JM, Barbarin A, et al. The impact of invariant NKT cells in sterile inflammation: the possible contribution of the alarmin/cytokine IL-33. Front Immunol. 2018;9:2308. 10.3389/fimmu.2018.02308 PMC6197076 30374349

[170] Mattner J. Natural killer T (NKT) cells in autoimmune hepatitis. Curr Opin Immunol. 2013;25:697–703. 10.1016/j.coi.2013.09.008 PMC4013545 24148235

[171] Kido M, Watanabe N, Okazaki T, Akamatsu T, Tanaka J, Saga K, et al. Fatal autoimmune hepatitis induced by concurrent loss of naturally arising regulatory T cells and PD-1-mediated signaling. Gastroenterology. 2008;135:1333–43. 10.1053/j.gastro.2008.06.042 18651994

[172] Horst AK, Neumann K, Diehl L, Tiegs G. Modulation of liver tolerance by conventional and nonconventional antigen-presenting cells and regulatory immune cells. Cell Mol Immunol. 2016;13:277–92. 10.1038/cmi.2015.112 PMC4856800 27041638

[173] Oikawa T, Takahashi H, Ishikawa T, Hokari A, Otsuki N, Azuma M, et al. Intrahepatic expression of the co-stimulatory molecules programmed death-1, and its ligands in autoimmune liver disease. Pathol Int. 2007;57:485–92. 10.1111/j.1440-1827.2007.02129.x 17610472

[174] Mühlbauer M, Fleck M, Schütz C, Weiss T, Froh M, Blank C, et al. PD-L1 is induced in hepatocytes by viral infection and by interferon-alpha and -gamma and mediates T cell apoptosis. J Hepatol. 2006;45:520–8. 10.1016/j.jhep.2006.05.007 16876901

[175] Aoki N, Kido M, Iwamoto S, Nishiura H, Maruoka R, Tanaka J, et al. Dysregulated generation of follicular helper T cells in the spleen triggers fatal autoimmune hepatitis in mice. Gastroenterology. 2011;140(1322–1333):e1321–5. 10.1053/j.gastro.2011.01.002 21237169

[176] Miyake Y, Yamamoto K, Matsushita H, Abe M, Takahashi A, Umemura T, et al. Multicenter validation study of anti-programmed cell death-1 antibody as a serological marker for type 1 autoimmune hepatitis. Hepatol Res. 2014;44:1299–307. 10.1111/hepr.12305 24506172

[177] van Beuge MM, Prakash J, Lacombe M, Post E, Reker-Smit C, Beljaars L, et al. Enhanced effectivity of an ALK5-inhibitor after cell-specific delivery to hepatic stellate cells in mice with liver injury. PLoS One. 2013;8:e56442. 10.1371/journal.pone.0056442 PMC3575413 23441194

[178] Bayer EM, Herr W, Kanzler S, Waldmann C, Meyer Zum Büschenfelde KH, Dienes HP, et al. Transforming growth factor-beta1 in autoimmune hepatitis: correlation of liver tissue expression and serum levels with disease activity. J Hepatol. 1998;28:803–11. 10.1016/s0168-8278(98)80230-4 9625315

[179] Budhu S, Schaer DA. Blockade of surface-bound TGF-β on regulatory T cells abrogates suppression of effector T cell function in the tumor microenvironment. Sci Signal. 2017;10:10. 10.1126/scisignal.aak9702 PMC5851440 28851824

[180] Holdener M, Hintermann E, Bayer M, Rhode A, Rodrigo E, Hintereder G, et al. Breaking tolerance to the natural human liver autoantigen cytochrome P450 2D6 by virus infection. J Exp Med. 2008;205:1409–22. 10.1084/jem.20071859 PMC2413037 18474629

[181] Pi L, Jorgensen M, Oh SH, Protopapadakis Y, Gjymishka A, Brown A, et al. A disintegrin and metalloprotease with thrombospondin type I motif 7: a new protease for connective tissue growth factor in hepatic progenitor/oval cell niche. Am J Pathol. 2015;185:1552–63. 10.1016/j.ajpath.2015.02.008 PMC4450322 25843683

[182] Gil-Farina I, Di Scala M. Transient Expression of Transgenic IL-12 in Mouse Liver Triggers Unremitting Inflammation Mimicking Human Autoimmune Hepatitis. J Immunol (Baltimore, Md: 1950). 2016;197:2145–56. 10.4049/jimmunol.1600228 27511737

