# Corrigendum to “The progress of autoimmune hepatitis research and future challenges”

**DOI:** 10.1515/med-2025-9993

**Published:** 2025-07-18

**Authors:** Yang Zhang, Dehe Zhang, Ling Chen, Jing Zhou, Binbin Ren, Haijun Chen

**Affiliations:** Graduate School of Zhejiang Chinese Medical University, Hangzhou, Zhejiang, China; Department of Infectious Diseases, Affiliated Jinhua Hospital, Zhejiang University School of Medicine, Jinhua, China

In the published article “Zhang Y, Zhang D, Chen L, Zhou J, Ren J, Chen H. The progress of autoimmune hepatitis research and future challenges. Open Med (Wars). 2023 Oct 30;18(1):20230823. doi: 10.1515/med-2023-0823. PMID: 38025543 PMCID: PMC10655690,” the affiliation of the first author is incorrect.

The department name “**Graduate Department of Zhejiang Chinese Medicine University**” in the first affiliation of the original article should be changed to “**Graduate School of Zhejiang Chinese Medical University, Hangzhou, Zhejiang, China.**”

